# Tailoring of the polarization-resolved second harmonic generation in two-dimensional semiconductors

**DOI:** 10.1515/nanoph-2024-0267

**Published:** 2024-07-31

**Authors:** Sotiris Psilodimitrakopoulos, Stepan Ilin, Lev E. Zelenkov, Sergey Makarov, Emmanuel Stratakis

**Affiliations:** Qingdao Innovation and Development Center, Harbin Engineering University, Qingdao, China; Foundation for Research and Technology-Hellas (FO.R.T.H), Heraklion, Crete, Greece; ITMO University, Saint Petersburg, Russia

**Keywords:** second harmonic generation, 2D materials, 2D chalcogenides, 2D perovskites, nanophotonics

## Abstract

Second harmonic generation is a non-linear optical phenomenon in which coherent radiation with frequency *ω* interacts with a non-centrosymmetric material and produces coherent radiation at frequency 2*ω*. Owing to the exciting physical phenomena that take place during the non-linear optical excitation at the nanoscale, there is currently extensive research in the non-linear optical responses of nanomaterials, particularly in low-dimensional materials. Here, we review recent advancements in the polarization-resolved second harmonic generation propertied from atomically thin two-dimensional (2D) crystals and present a unified theoretical framework to account for their nonlinear optical response. Two major classes of 2D materials are particularly investigated, namely metal chalcogenides and perovskites. The first attempts to tune and control the second harmonic generation properties of such materials via the application of specific nanophotonic schemes are additionally demonstrated and discussed. Besides presenting recent advances in the field, this work also delineates existing limitations and highlights emerging possibilities and future prospects in this field.

## 1 Introduction

Second harmonic generation (SHG) was first demonstrated in 1961 by Franken et al. in crystalline quartz [1]. SHG is a non-linear optical (NLO) phenomenon in which coherent radiation with frequency *ω* interacts with a non-centrosymmetric material and produces coherent radiation at frequency 2*ω*. Nowadays, there is extensive research for microscale and nanoscale materials that show NLO optical responses, namely low-dimensional NLO materials. Numerous studies are performed in the SHG optical properties of low-dimensional materials [2]. Such materials are key to developing future photonic and optoelectronic devices that offer high SHG efficiency, low energy usage, and very small size. Recently, two-dimensional (2D), atomically thin, materials have shown great potential for use in the development of such optoelectronic devices. To date, SHG is identified in numerous such 2D crystals and several reviews are addressing their NLO properties [3–8].

Nonlinear optics encompasses a wide range of applications, including photon generation, manipulation, transmission, detection, and imaging. Nonlinear optical phenomena occur in the presence of intense light, and they have been a subject of intense study of numerous NLO effects. NLO plays a crucial role in various applications such as laser frequency conversion, ultrafast pulsed laser generation, terahertz (THz) wave generation, optical switches, photodetectors, and optical modulators [9–13]. To facilitate these applications, the development of nonlinear optical materials is essential [14]. The bulk NLO materials exhibit issues with a low nonlinear coefficient and suboptimal conversion efficiency [11]. This limitation is crucial when integrating them into miniaturized photonic and optoelectronic devices.

Over the past few decades, there has been a growing interest in 2D materials as NLO materials [2–13]. For more than a decade, 2D materials have been a hot topic owing to their unique material features, ultra-thin scale, and outstanding linear and nonlinear optical capabilities that make them ideal for a wide variety of device applications. After discovering graphene in 2004, many efforts have been made to uncover the SHG physics in 2D materials. Continuous advancements in preparation methods and growth techniques for 2D materials have enabled high-quality and large-scale production, which provides favorable conditions for commercial use [14].

In 2009, the graphene’s exceptional saturable absorption characteristics were presented [15] Since then, various research teams have experimentally confirmed the optical nonlinearity of 2D materials and demonstrated numerous impressive nonlinear optical devices based on 2D materials [2–12], [16], [17], [18], [19]. In the last decade, transition metal dichalcogenides (TMDs) have gained significant attention because of their unique physical properties, such as high exciton binding energies [20], [21], direct bandgap in the visible and IR ranges [22], [23], and naturally occurring intrinsic valley polarization [24]. In 2013, researchers began to investigate SHG in TMDs [2–13]. The research of SHG is also conducted in various 2D materials. SHG in 2D materials is a result of confining electrons within an atomically thin plane, which induces a pronounced second-order nonlinear optical response. SHG research on TMDs attracted the attention of many researchers due to the following features. First, their 2D crystal structure is non-centrosymmetric enabling SHG processes, which is not the case in graphene [9]. Second, there are numerous possibilities for TMD homo- and hetero-structures with strong and tunable SHG responses [2–13]. Last but not least, SHG in TMDs is a rapidly developing area with a lot of novel physics, such as valley selective SHG, controlling the SHG in bilayers by tuning the twist-angle, Moiré nanostructures and polaritons [2–13], [25]. Furthermore, it has been demonstrated that the excitonic states in TMDs are highly correlated with the SHG response delivered by TMDs and their heterostructures [26]. The integration of resonant nanostructures with TMDs has also attracted a great deal of attention as they enhance the SHG from TMDs [27]. In addition, several other factors, including strain, electric gating [25], excitonic resonance, unusual optical resonances [28], phase and edge modulation [29], also have been used to manipulate the SHG in TMDs.

Here, we review recent advancements in the polarization-resolved SHG (P-SHG) signals from such atomically thin crystals, placing emphasis on the fundamental unified theoretical framework of nonlinear optics for P-SHG measurements that is applied to the latest literature reports. In particular, we investigate recent advancements on how the P-SHG technique is used in two major classes of 2D materials, namely metal chalcogenides and perovskites, as an effective characterization method. Furthermore, first attempts to tune and control the SHG properties via nanophotonic schemes are presented and discussed.

## 2 Second harmonic generation in 2D transition metal dichalcogenides and 2D metal monochalcogenides

The field of 2D materials initiated in 2004, when Konstantin Novoselov and Andre Geim used Scotch tape to strip, for the first time, monoatomic thickness graphene from graphite [30]. Shortly after the discovery of graphene, the 2D materials family grew bigger with the introduction of the 2D transition metal dichalcogenides and the introduction of 2D metal monochalcogenides.

Group IV metal monochalcogenides, denoted by MX with M = Sn, Ge and X = S, Se, are a class of layered, orthorhombic (mm^2^ point symmetry group), semiconducting 2D materials attracting significant interest [31]–[33]. They are known as phosphorene analogues [31]–[34], since they share similar puckered or wavy lattice structures with phosphorene, a 2D format of black phosphorus [35], [36]. The in-plane structural anisotropy of MXs, with puckered structure along the armchair direction [33] induces in-plane anisotropic physical properties [31]–[33], [37], [38]. A plethora of properties have been reported to exhibit in-plane anisotropic response, including carrier mobility [37], optical absorption, reflection, extinction, refraction [38] and Raman spectral behavior [31]. The in-plane anisotropic response is exhibited along the distinguished in-plane armchair and zig-zag crystallographic directions, offering an additional degree of freedom in manipulating their properties [31]–[33], [37], [38].

On the other hand, the 2D TMDs are direct bandgap semiconductors and they follow the formula MX_2_, where M is: Mo or W, and X counts for: S, Se, or Te. These 2D TMDs exhibit diverse band structures and demonstrate distinctive optical, electrical, and mechanical characteristics. They offer a basis for ongoing advancements across various fields of research and hold significant potential for the development of ultra-thin, low-power transistors [39]. Moreover, 2D TMDs show inequivalent valleys at the corners of the Brillouin zone, leading to new valley degree of freedom [40]–[42]. Additionally, the strong quantum confinement at the atomic level renders the SHG response of 2D TMDs highly sensitive to the number of layers, ranging from one single layer to the bulk form [43]–[45]. In 2D crystals the atoms are arranged into a periodic pattern that is described by various types of crystal symmetries [46]. The crystal symmetry is essential in determining the physical properties of materials. Therefore, a key objective in the study of 2D materials is to determine the crystal symmetry and identify the armchair direction of the crystal. In contrast to conventional X-ray diffraction (XRD) and transmission electron microscopy (TEM) methods, the all-optical second harmonic generation (SHG) offers minimally-invasive means to examine 2D materials, bypassing complex sample preparation processes (e.g. transfer in a TEM grid etc.). SHG is a second-order NLO process that occurs in non-centrosymmetric materials [47]. For example, TMD monolayers lack inversion symmetry and produce strong SHG signals [48], [49]. The TMD monolayers with in plane threefold rotational symmetry, belong to the D_3h_ crystal symmetry group. In Table 1 typical *χ* ^(2)^ values are presented [2].

**Table 1:** Typical *χ* ^(2)^ values for 2D materials [2].

2D Material	Crystal symmetry	*χ* ^(2)^ (10^−12 m/V^)	SHG wavelength	Substrate	Thickness	Fabrication method
SnS	mm^2^	1.37	450 nm	MgO	∼30 nm	MBE
MoS_2_	D_3h_	105	405 nm	SiO_2_/Si	1L	Exfoliation
MoSe_2_	D_3h_	50	810 nm	SiO_2_/Si	1L	CVD
WS_2_	D_3h_	4500	415 nm	SiO_2_/Si	1L	Exfoliation
WSe_2_	D_3h_	100	775 nm	SiO_2_/Si	1L	Exfoliation
MoTe_2_	D_3h_	2500	775 nm	SiO_2_/Si	1L	Exfoliation

Quantifying and modelling the SHG signals intensity in 2D materials requires accounting for various physical phenomena. For example, the SHG intensity from 2D materials is affected by the boundary conditions, due to the confinement of electrons that can significantly alter the electronic states and, consequently, the nonlinear optical response. For example, grain boundaries may exhibit electronic and optical properties completely different from the rest of the material [9]. On the other hand, resonant enhancement of SHG can occur when the incident or second harmonic frequency matches the electronic or phononic resonances of the material. This resonance can lead to a substantial increase in SHG intensity. For example, SHG resonance at about 1,310 nm is observed on the atomic edges of monolayer MoS_2_ [50]. The nonlinear optical susceptibility is significantly enhanced when the real energy level is in the vicinity of virtual states that are involved in the nonlinear optical process as density of states dramatically increase [25]. Additionally, the choice of substrate can affect the SHG signal due to changes in the local field enhancement and interference effects. For example, a significant enhancement of SHG can be achieved by placing a monolayer of 2D material on a photonic moiré superlattice made from dielectric materials. The local field enhancement at the resonance modes of the moiré superlattice can dramatically boost the SHG response. For instance, a MoS_2_ monolayer on a photonic moiré superlattice showed an SHG conversion efficiency orders of magnitude larger than that from a monolayer on a flat dielectric slab [51]. The choice of substrate material affects the SHG signal. For example, an SHG enhancement was observed when comparing fluorine-doped tin oxide and glass as substrates. This indicates that the substrate’s optical properties can significantly influence the SHG intensities [52]. Additionally, metal nanostructures can enhance SHG through surface plasmon resonance. This enhancement occurs because the central symmetry at the interface of two centrally symmetric media is disrupted, leading to the generation of a second harmonic signal at the interface layer [53]. Moreover, the use of high numerical aperture (NA) objectives focuses the incident light into a tight spot, increasing the local field intensity and enhancing the SHG signal. However, high NA objectives can also introduce aberrations and distortions in the beam profile altering the field distribution and affecting the SHG measurement. These distortions can affect the uniformity and intensity of the SHG signal across the 2D material [54]. In summary, quantifying the SHG intensity in 2D materials requires a comprehensive understanding of the material’s properties and the experimental conditions.

SHG signals are coherent with well-defined polarizations, thus polarization-resolved measurements enable the extraction of additional information than SHG intensity only measurements [55], [56]. In particular, P-SHG measurements provide information about the direction of the crystal armchair direction and crystal homogeneity [49], [57–63] grain boundaries [48], [64] stacking sequence and twist-angle [65], [66], number of layers [59], [62] in plane anisotropy [67], valley polarization [68], [69] and strain [70], [71].

### 2.1 Layer-dependent SHG in 2D transition metal dichalcogenides

**Figure 1:**
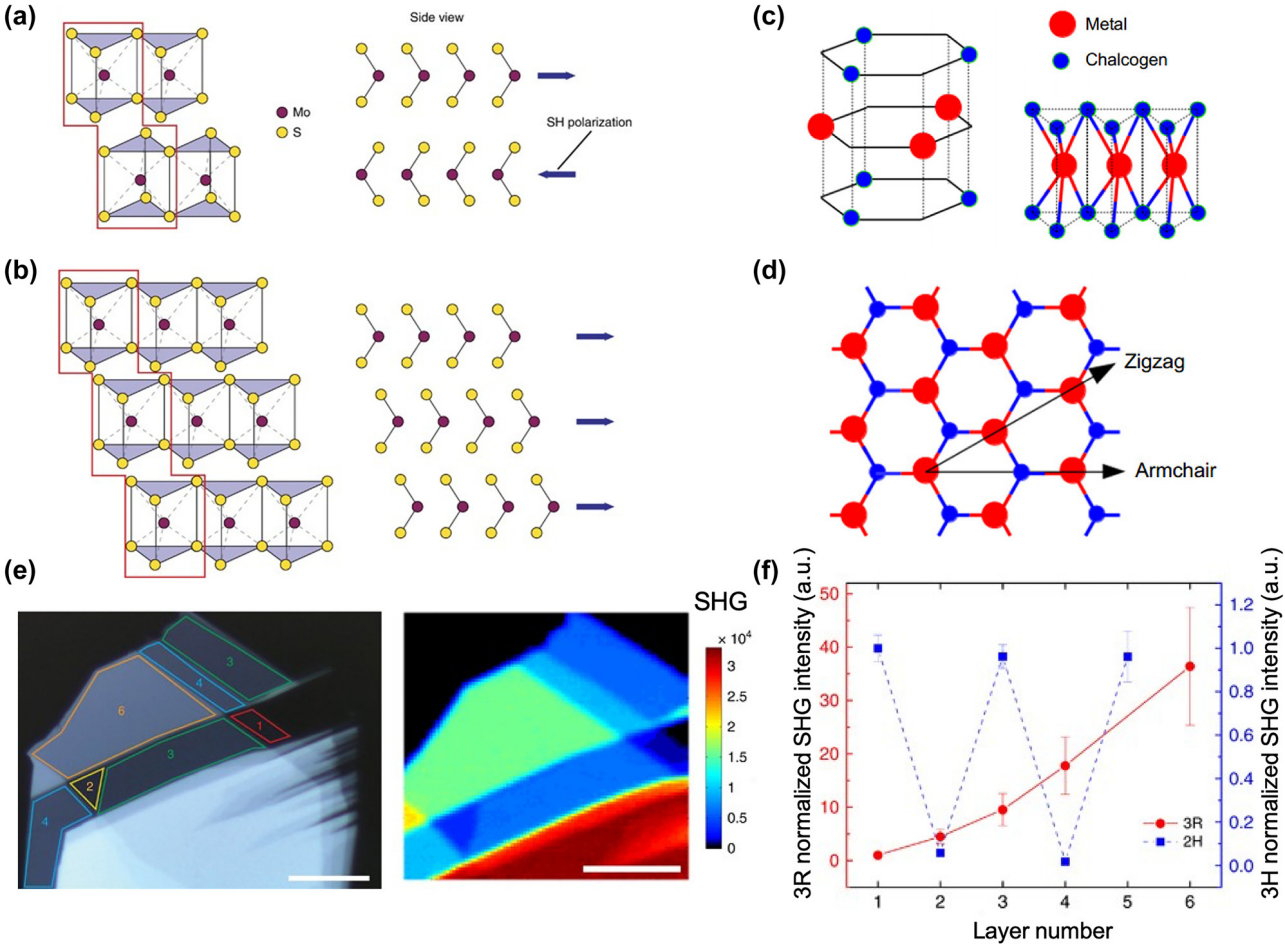
Layer-dependent SHG in 2D TMDs. (a) Schematic of the crystal structure of 2H-MoS_2_ (two-layer unit cell). The side projection shows the flipped orientation of each layer and the anti-parallel orientation of the SHG dipoles that cancel the SHG. (b) Crystal structure of 3R-MoS_2_ (three-layer unit cell). The layers are oriented in such a way that the dipoles are parallel allowing for production of SHG. (c) Schematic representation of the structure of 3R-TMDs, containing three sublattices, with a plane of metal atoms being hexagonally packed between two planes of chalgogen atoms. (d) Top view where the armchair and zig-zag directions are drawn. Non-centrosymmetry and thus the production of SHG originates from the interchange of halogen/metal atoms in the armchair crystal direction. (e) Optical image of the 3R-MoS2 crystal, where the number of layers are seen on top of the corresponding areas and mapping of the produced SHG. (f) SHG intensity of the 3R and 2H-MoS_2_ normalized to the respective single-layer intensity. The dependence of the SH intensity is squared with relation to the layer number in the 3R crystal, while it oscillates with number of layers in the 2H crystal. (a), (b), (e), (f) are reprinted with permission from [60]. Copyright 2016. Springer Nature. (c), (d) are reprinted with permission from [62]. Copyright 2020. Institute of Optics and Electronics, Chinese Academy of Sciences.

The MX_2_ crystal comprises two honeycomb sublattices – one comprising M atoms and the other X atoms – shifted relative to each other; therefore, in its bulk form, the 2H-MX_2_ crystals are inversion symmetric (for an even number of layers), belonging to the D_6h_ symmetry group, characteristic of the Bernal-stacked trigonal prismatic structure (Figure 1(a)) [60].

However, this symmetry is broken for an odd number of layers due to stacking termination along the *c*-axis, resulting in non-centrosymmetric unit cell (3R stacking Figure 1(b) [60]) that give rise to SHG. In the case of odd layers unit cell, an individual layer of M atoms with threefold symmetry is hexagonally packed between two trigonal atomic layers of X atoms, resulting in a hexagonal honeycomb lattice, where the M and X atoms are located at alternate corners of the hexagon (Figure 1(c) [62]). Therefore, the MX_2_ monolayer belongs to the D_3h_ point symmetry group, with broken inversion symmetry along the armchair direction (Figure 1(d) [62]). The lack of inversion symmetry in the monolayer results in SHG when an intense field is incident on the crystal [57] (Figure 1(e)). For nanoflakes with even layer number, the SHG signals from adjacent layers are cancelled out. Hence, high contrast of SHG intensity between odd and even numbers can be used to characterize parity of number of layers. For odd layers of MX_2_ with finite SHG, the exact layer number is further identified by intensity variation of SHG with number of layers [58] (Figure 1(f)). In particular, the induced non-linear dipoles allow for constructive interference of SHG, resulting in quadratic dependent SHG intensity with the number of layers [60] (Figure 1(f)). On the other hand, in 1T′ phase form, the layer dependency of SHG is opposite to that in 2H phase, i.e. even layers of 1T′ crystal produce significant SHG while odd layers with inversion symmetry show negligible SHG [61]. Therefore, the stacking sequence of 2D materials plays an important role in the SHG intensity.

### 2.2 Pixel-wise mapping of 2D TMDs armchair directions; polarization-resolved SHG and raster-scanning

**Figure 2:**
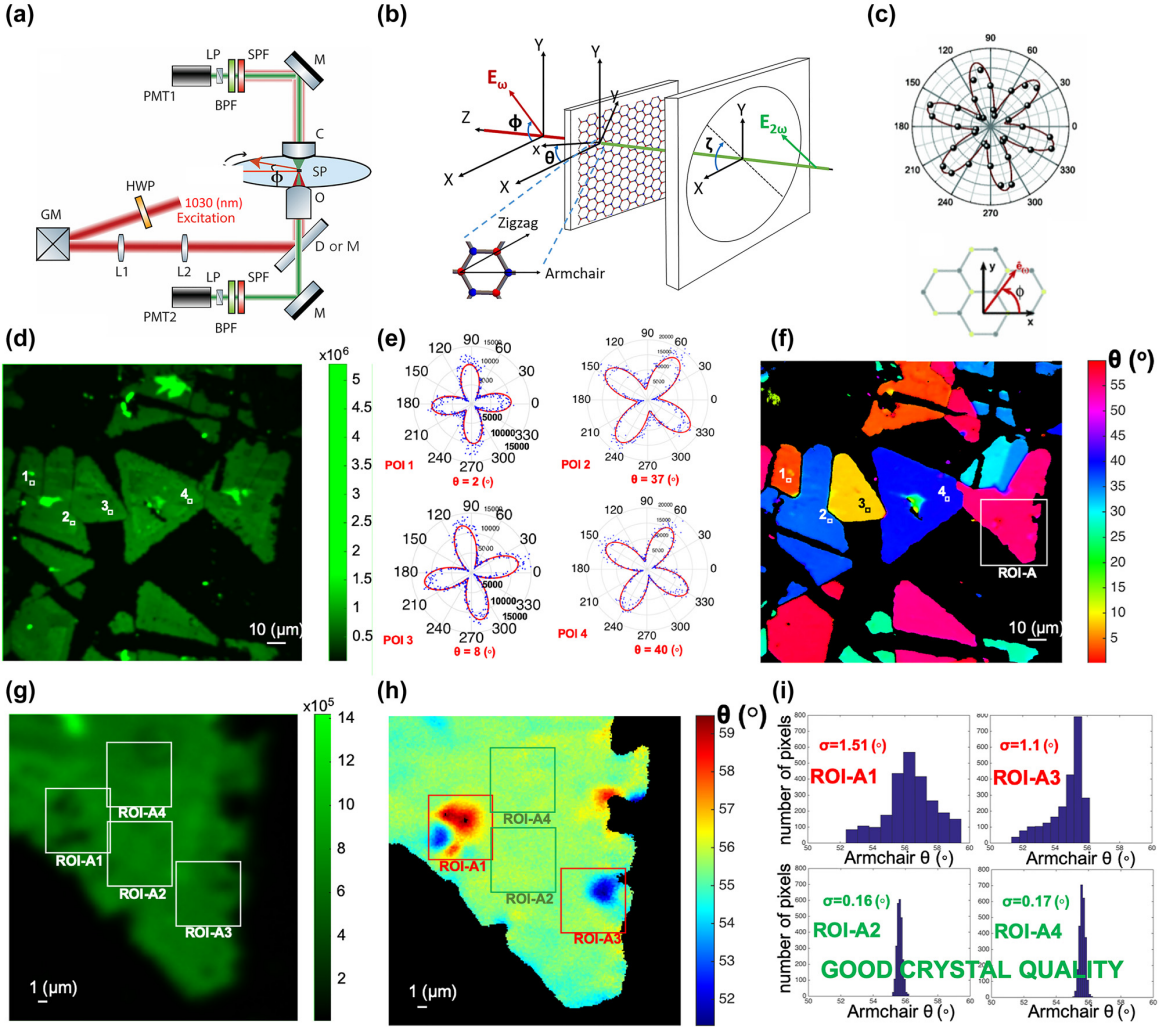
Pixel-wise mapping of 2D TMDs armchair directions identifies 2D crystal imperfections. (a) Typical raster-scanning P-SHG experimental setup; HWP, half-waveplate; GM, galvanometric mirrors; L1,2, lenses; D or M, dichroic (for epi detection) or mirror (for forward detection); O, objective lens; SP, sample plane; C, condenser; M, mirror; SPF, short-pass filter to cut-off the laser; BPF, bandpass filter for SHG; LP, linear polarizer; PMT1,2, photomultiplier tubes. The excitation linear polarization is rotated with an angle *φ*, while the linear polarizer is rotated with *ζ* = *φ* (6 lobes polar diagrams) or is fixed, e.g. at *ζ* = 0° or *ζ* = 90° (4 lobes polar diagrams). (b) Coordinate system describing the experimental measurements and introducing relevant angles with respect to the laboratory frame (*XYZ*); *φ*: angle between excitation linear polarization *E* ^*ω*^ and *X*-laboratory axis; *θ*: angle between armchair direction −*x* and the *X*-lab axis; *ζ*: angle between linear polarizer axis and *X*-axis. The crystal structure and armchair direction of a TMD crystal are shown (top view). (c) Experimental P-SHG data from a MoS_2_ monolayer for *ζ* = *φ* (6 lobes polar diagram) and fitting using Eq. (6). Top view of the MoS_2_ crystallographic orientation with respect to the incident laser polarization. (d) SHG image of CVD grown WS_2_ 2D flakes. Four pixels of interest (POIs) are marked. (e) Experimentally retrieved P-SHG polar diagrams from the four POIs. The rotation of the polar diagrams corresponds to different armchair directions *θ*. (f) Mapping of armchair orientations *θ* over a large sample area. The POIs shown correspond to the same positions as in (d). (g) Magnified view of ROI-A SHG intensity. (h) The mapping of armchair directions reveals grains of different crystal orientations, not seen in the SHG intensity image. (i) Image histograms showing the distribution of armchair orientations inside ROIs-A1-4. The crystal quality is reflected in the standard deviation (*σ*) of the mean armchair direction, <*θ*>. Small *σ* values are indicative of good crystal quality. (a), (b), (d), (e), (f), (g), (h), (i) are reproduced with permission from [59]. Copyright 2018. Springer Nature. (c) is reprinted with permission from [57]. Copyright 2013. American Physical Society.

Polarization-resolved SHG (P-SHG) establishes a relation with the direction of the 2D material armchair crystallographic axes [58]. The optical setup for P-SHG measurements is based on controlled excitation polarization and controlled detection of the polarization of the SHG signals, while raster-scanning is performed using galvanometric mirrors (Figure 2(a)) or moving piezoelectric stages [2–8].

In order to describe the interaction of an excitation field with a 2D MX_2_ and the subsequent production of SHG, we use the following formalism [59], [62].

Two coordinate systems are schematically shown in Figure 2(b): the laboratory frame (*X*, *Y*, *Z*) and the crystal coordinates (*x*, *y*, *z*), where *z* ≡ *Z*. The laser is normally incident on the crystal, and linearly polarized along the sample plane, at an angle *φ* with respect to *X* laboratory axis. By rotating the half-waveplate, we vary the orientation of the excitation linear polarization, and record the SHG emerging from the sample as function of the polarization angle *φ*. The *x* axis is taken parallel to the armchair direction of the crystal and at an angle *θ* from *X* (Figure 2(b)).

The excitation field after passing the half-wave retardation plate can be expressed in laboratory coordinates by the vector $\left(\begin{array}{c} E_{0}\cos\varphi \\ E_{0}\sin\varphi \end{array}\right)$ , where *E*_0_ is the amplitude of the electric field. The expression of this vector in the coordinates system of the 2D crystal can be derived by multiplying the excitation field with the rotation matrix

$$\left(\begin{array}{cc} \cos\theta & \sin\theta \\ -\sin\theta & \cos\theta \end{array}\right),\text{giving}\;E^{\omega }=\left(\begin{array}{c} E_{0}\cos\left(\varphi -\theta \right)\\ E_{0}\sin\left(\varphi -\theta \right) \end{array}\right).$$

As we saw above, the 3R TMD monolayers MX_2_ belong to the D_3h_ symmetry group [48], [49], [57–64] and their intrinsic second-order nonlinear susceptibility tensor, $\chi_{\text{int}}^{\left(2\right)}$ features the following non-zero elements [49], [57]:

$$\chi_{\mathrm{xxx}}=-\chi_{\mathrm{xyy}}=-\chi_{\mathrm{yxy}}=\chi_{\text{int}}^{\left(2\right)}$$

As a result, the 2nd order induced polarization is expressed as [59], [62]:

(1) $$\left(\begin{array}{c} P_{x}^{2\omega }\\ P_{y}^{2\omega }\\ P_{z}^{2\omega } \end{array}\right)=\varepsilon_{0}\chi_{\text{int}}^{\left(2\right)}\left(\begin{array}{cccccc} 1 & -1 & 0 & 0 & 0 & 0\\ 0 & 0 & 0 & 0 & 0 & -1\\ 0 & 0 & 0 & 0 & 0 & 0 \end{array}\right)\left(\begin{array}{c} E_{x}^{\omega }E_{x}^{\omega }\\ E_{y}^{\omega }E_{y}^{\omega }\\ E_{z}^{\omega }E_{z}^{\omega }\\ 2E_{y}^{\omega }E_{z}^{\omega }\\ 2E_{x}^{\omega }E_{z}^{\omega }\\ 2E_{x}^{\omega }E_{y}^{\omega } \end{array}\right),$$

where, *ɛ*_0_ is the permittivity of the free space. Considering that the pump laser beam is polarized along the sample plane, i.e., $E_{z}^{\omega }=0$ , Eq. (1) is reduced to:

(2) $$\left(\begin{array}{c} P_{x}^{2\omega }\\ P_{y}^{2\omega } \end{array}\right)=\varepsilon_{0}\chi_{\text{int}}^{\left(2\right)}\left(\begin{array}{c} {\left(E_{x}^{\omega }\right)}^{2}-{\left(E_{y}^{\omega }\right)}^{2}\\ -2E_{x}^{\omega }E_{y}^{\omega } \end{array}\right).$$

We then transform Eq. (2) back to laboratory coordinates by multiplying with the rotation matrix $\left(\begin{array}{cc} \cos\theta & -\sin\theta \\ \sin\theta & \cos\theta \end{array}\right)$ , obtaining:

(3) $$\left(\begin{array}{c} P_{X}^{2\omega }\\ P_{Y}^{2\omega } \end{array}\right)=\varepsilon_{0}\chi_{\text{int}}^{\left(2\right)}\left(\begin{array}{c} \cos\left(3\theta -2\varphi \right)\\ \sin\left(3\theta -2\varphi \right) \end{array}\right).$$

By using a rotating polarizer in front of the detector, we collect the components of the SHG field, described by multiplying with the matrix $\left(\begin{array}{cc} \cos^{2}\zeta & \sin\zeta \cos\zeta \\ \sin\zeta \cos\zeta & \sin^{2}\zeta \end{array}\right)$ . Here, *ζ* is the angle of the transmission axis of the polarizer with respect to *X*-axis.

Then, the intensity of the SHG field is described by [59], [62]:

(4) $$I^{2\omega }=A\cos^{2}\left(\zeta -3\theta +2\varphi \right),$$

where *A* is a multiplication factor depending on $\chi_{\text{int}}^{\left(2\right)}$ and the amplitude of the excitation field.

For rotating polarizer with an angle *ζ* = *φ*, i.e. rotating polarizer always parallel to the direction of the excitation polarization *φ*, the SHG intensity reads [49], [57]:

(5) $$I^{2\omega }=A\cos^{2}3\left(\varphi -\theta \right).$$

The above experimental configuration provides 6 lobes P-SHG polar diagrams (Figure 2(c)) [57], that rotate for different armchair orientations *θ*.

For *ζ* = 0° or *ζ* = *π*/2 i.e. fixed polarizer parallel to *X* or *Y* laboratory axes, respectively, the SHG intensity reads [59], [62]:

(6) $$I_{X}^{2\omega }=A\text{co}{\text{s}}^{2}\left[3\theta -2\varphi \right]$$

and

(7) $$I_{ϒ}^{2\omega }=A\text{si}{\text{n}}^{2}\left[3\theta -2\varphi \right].$$

The result of the experimental configuration described by Eq. (6) (fixed analyzer at *ζ* = 0°) demonstrates a four-lobe polar diagram (Figure 2(e)), that rotates for different armchair orientations *θ*. The armchair directions differing by 60° produce the same polar diagrams (that is, the armchair direction can be determined modulo 60°), reflecting the threefold rotational symmetry of the MX_2_ crystal (that is, the fact that there are three equivalent armchair axes).

By fitting pixel-by-pixel the P-SHG experimental data (Figure 2(d)) to Eq. (6), the armchair direction *θ* for every point of the 2D crystals is acquired (Figure 2(f)) [59]. This pixel-wise mapping of the armchair directions provides the means for evaluating crystal homogeneity and thus crystals quality. By choosing a region of interest (ROI) in the crystal (Figure 2(g) and (h)), the armchair directions distribution is acquired, described by its mean and standard deviation *σ* (Figure 2(i)). Then *σ* is used as a quality criterion [59]. A broad distribution of armchair directions in the ROI indicates the presence of defects that affect the crystal structure and homogeneity, while a narrow distribution indicates a uniform crystal structure. Thus, the narrower the distribution of armchair directions is, the better the crystal quality is [59].

### 2.3 Polarization-resolved SHG measurements of twist-angles in artificial TMD homo/heterobilayers and multi-layer structures

Apart from single materials, the atomically thin 2D layers can stack into van der Waals heterostructures [72]. Nowadays, 2D heterostructures can be designed with arbitrary angles hence result in large variance of symmetries [73], [74]. Such variety leads to engineering of materials’ properties. For example, the band structure and optical properties of twisted bilayer MoS_2_ vary with the twisted angle [75]. Thus, it is of great importance to identify and precisely control the interlayer stacking angles of heterostructures [76].

**Figure 3:**
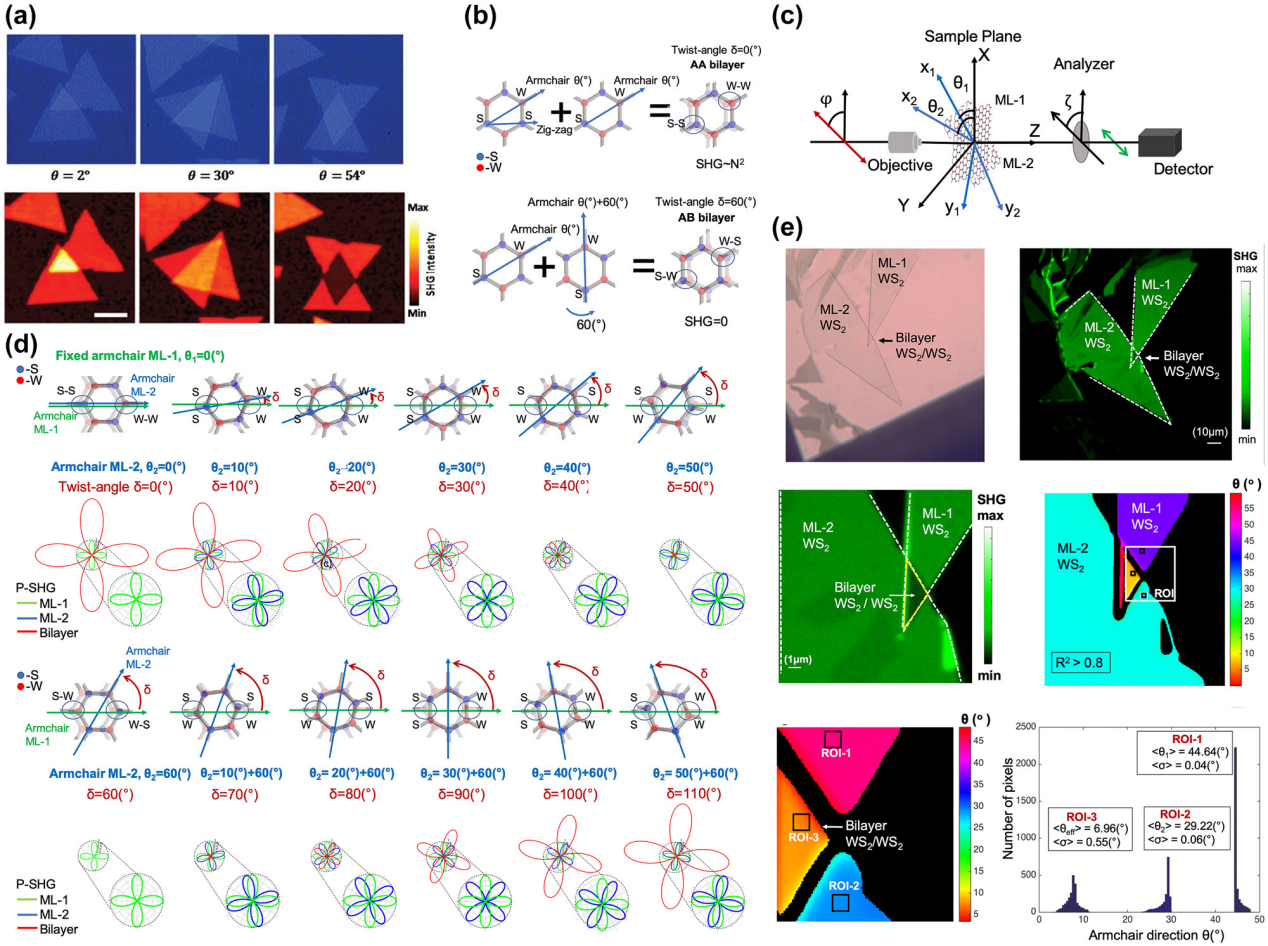
Twist-angle mapping in 2D TMD bilayers. (a) CCD image (up) and SHG intensity mapping (down) of MoS_2_ bilayers with different stacking angles of *θ* = 2°, 30° and 54°. (b) Schematic representation of the top view in the atomic configuration of a 2D WS_2_ bilayer, for the AA stacking sequence. Atoms of W–W or S–S are on top of each other and the SHG signals depend quadratically on the number *N* of the monolayers, (SHG ∼ *N* ^2^). In the atomic configuration for the AB stacking sequence (twist-angle = 60°) the alternating W–S atoms are on top of each other and the SHG signals cancel (SHG = 0), because centrosymmetry is restored. (c) Coordinate system of the experimental configuration used for P-SHG imaging. *θ*_1_ and *θ*_2_ denote the armchair directions of monolayer (ML) −1 and ML −2, respectively. *φ* indicates the direction of the excitation linear polarization and *ζ* the axis of the analyzer. In our experiments *ζ* is fixed to 0° which greatly simplifies the experimentally retrieval of the P-SHG polar diagrams. (d) Numerical simulation showing the effect of twist-angle in the polar diagrams of P-SHG. (e) Experimental extraction of *θ*_eff_ from the bilayer region. (a) is reproduced with permission from [65]. Copyright 2014. American Chemical Society. (b)–(e) are reproduced with permission from [79]. Copyright 2021. Springer Nature.

Considering the fabrication of a 2D heterostructure with specific requirement of stacking angle, the knowledge of crystal orientation is required. Designed vertical stacking of TMDs heterostructures with specific twist-angles creates new material system with tailored properties [77], [78]. Given the atomic phase-matching condition, SHG from the 2D heterostructure can be regarded as a coherent superposition of the SHG fields from the individual layers [65], [66], [79], with a phase difference depending on the stacking angle (Figure 3(a)) [65]. A recent work on modeling twist-angle in MoS_2_ was demonstrated with an open source package [80].

Hsu et al. [65] studied the SHG interference from artificially stacked TMD bilayers and showed that the total intensity $I_{N}^{2\omega }$ recorded in the bilayer region can be expressed as:

(8) $$\begin{array}{ll} I_{N}^{2\omega } & ={\left|\vec{E_{N}^{2\omega }}\right|}^{2}={\left|\overset{N}{\underset{i=1}{\sum }}\vec{E_{N}^{2\omega }}\right|}^{2}\\ & =\overset{N}{\underset{i=1}{\sum }}I_{i}^{2\omega }+\overset{N}{\underset{\begin{array}{c} i,j\\ \left(i\neq j\right) \end{array}}{\sum }}\sqrt{I_{i}^{2\omega }I_{j}^{2\omega }}\cdot \cos\left(3\delta_{ij}\right), \end{array}$$

with $I_{i}^{2\omega }$ the intensity of the *i*th layer, and *δ*_*ij*_ the twist-angle between layers *i* and *j*, *δ*_*ij*_ = *θ*_*i*_ − *θ*_*j*_.

For *N* = *2*, the SHG intensity of the bilayer (BL) is given by [65]:

(9) $$I_{\left(BL\right)}^{2\omega }=I_{1}^{2\omega }+I_{2}^{2\omega }+2\sqrt{I_{1}^{2\omega }I_{2}^{2\omega }}\cdot \cos\left(3\delta \right).$$

Furthermore, for layers of equal SHG intensity ( $I_{1}^{2\omega }=I_{2}^{2\omega }=I_{ML}$ ) at zero twist-angle (*δ* = 0), we obtain, $I_{\left(BL\right)}^{2\omega }=4I_{\left(ML\right)}^{2\omega }$ , i.e. the well-known result that SHG intensity scales quadratically with layer number (Figure 3(b)).

The above analysis [65] is based on SHG intensities measurements only (Figure 3(a)), i.e. at a fixed excitation polarization and without the use of an analyzer. Using Eq. (9) two intensity measurements, one in the monolayer and one in the bilayer regions can provide twist-angle values modulo 60°.

Nevertheless, it is demonstrated that P-SHG measurements, i.e. rotation of the excitation linear polarization, provides a more detailed description than the intensity only measurements and reveals the twist-angle values modulo 120° [66], [79].

In the P-SHG experimental scenario, as before, the measurement utilizes rotation of linear excitation polarization in an angle *φ* with respect to the *X* lab axis (Figure 3(c)).

The SHG of *N* number of such one-armchair layers, built with a stacking angle *δ*_*ij*_, for the case of fixed analyzer at *ζ* = 0° and rotating linear polarization in an angle *φ*, is then described by [66], [79]:

(10) $$I_{N}^{2\omega }={\left|\overset{N}{\underset{i=1}{\sum }}A_{i}\cos\left(3\theta_{i}-2\varphi \right)\right|}^{2},$$

where *θ*_*i*_ are the armchair directions of each individual flake. In the case of two monolayers (*N* = 2) reduces to [66], [79]:

(11) $$I_{\left(BI\right)}^{2\omega }={\left[A_{1}\cos\left(3\theta_{1}-2\varphi \right)+A_{2}\cos\left(3\theta_{2}-2\varphi \right)\right]}^{2},$$

where *θ*_1_ and *θ*_2_ are the armchair directions of each individual flake.

One can therefore employ Eq. (11) to deduce the real armchair orientation of a second layer that resulted to the total SHG from both layers.

Assuming *A*_1_ = *A*_2_ (layers of equal SHG i.e. WS_2_/WS_2_ homobilayer) in Figure 3(d) are presented the theoretical P-SHG modulations of the two monolayers, as well as the SHG modulation of the product of their interference in their overlapping region, for several armchair twist-angles *δ* [79].

The effective armchair direction *θ*_eff_ in the overlapping region of the two TMD monolayers that describes the total SHG intensity produced by the 2 monolayers is given by [66], [79] as:

(12) $$I_{\left(BI\right)}^{2\omega }={\left[A_{\text{eff}}\cos\left(3\theta_{\text{eff}}-2\varphi \right)\right]}^{2}$$

where

(13) $$A_{\text{eff}}=2Acos\frac{3}{2}\delta ,$$

where *δ* = *θ*_1_ − *θ*_2_ is the twist-angle, between the monolayers and

(14) $$\theta_{\text{eff}}=\frac{\theta_{1}+\theta_{2}}{2}.$$

This means that the P-SHG modulation emerging from a bilayer region consisting of two TMD monolayers, at twist angle *δ* = *θ*_1_ − *θ*_2_, behaves as if it was the P-SHG modulation of a single monolayers with armchair direction *θ*_eff_. The *θ*_eff_ is extracted experimentally from the P-SHG polar obtained from the bilayer region [66], [79].

Additionally, as it is noted in Figure 3(c), the SHG signals originating from the bilayers regions with twist-angles of 10(°) and 110(°), 20(°) and 100(°), 30(°) and 90(°), and 40(°) and 80(°), are of equal SHG intensities. Nevertheless, the P-SHG interference polar diagrams from the bilayers’ regions (red lines in Figure 3(d)) for the twist-angles of 10(°) and 110(°), 20(°) and 100(°), 30(°) and 90(°), and 40(°) and 80(°), are different, thus P-SHG is able to identify and discriminate twist-angles that produce equal SHG intensities from the bilayer regions. Since *θ*_1_, *θ*_2_ ∈ [0°, 60°] (i.e. modulo 60°) we have that *δ* ∈ [−60°, 60°] (i.e. modulo 120°) and *θ*_eff_ ∈ [0°, 60°] (i.e. modulo 60°) [66]. From Figure 3(d) it is evident that by using P-SHG measurements and by controlling the twist-angle between two monolayers allows for full control of intensity and polarization of the produced SHG from the bilayer region.

### 2.4 Single-scan calculation of twist angle in 2D TMD heterobilayers

In the case of TMD monolayers the armchair direction *θ* could also be determined by combining the Eqs. (6) and (7), as [62], [63]:

(15) $$\theta =\frac{1}{3}\left(2\varphi +tan^{-1}\sqrt{\frac{I_{ϒ}^{2\omega }}{I_{X}^{2\omega }}}\right).$$

**Figure 4:**
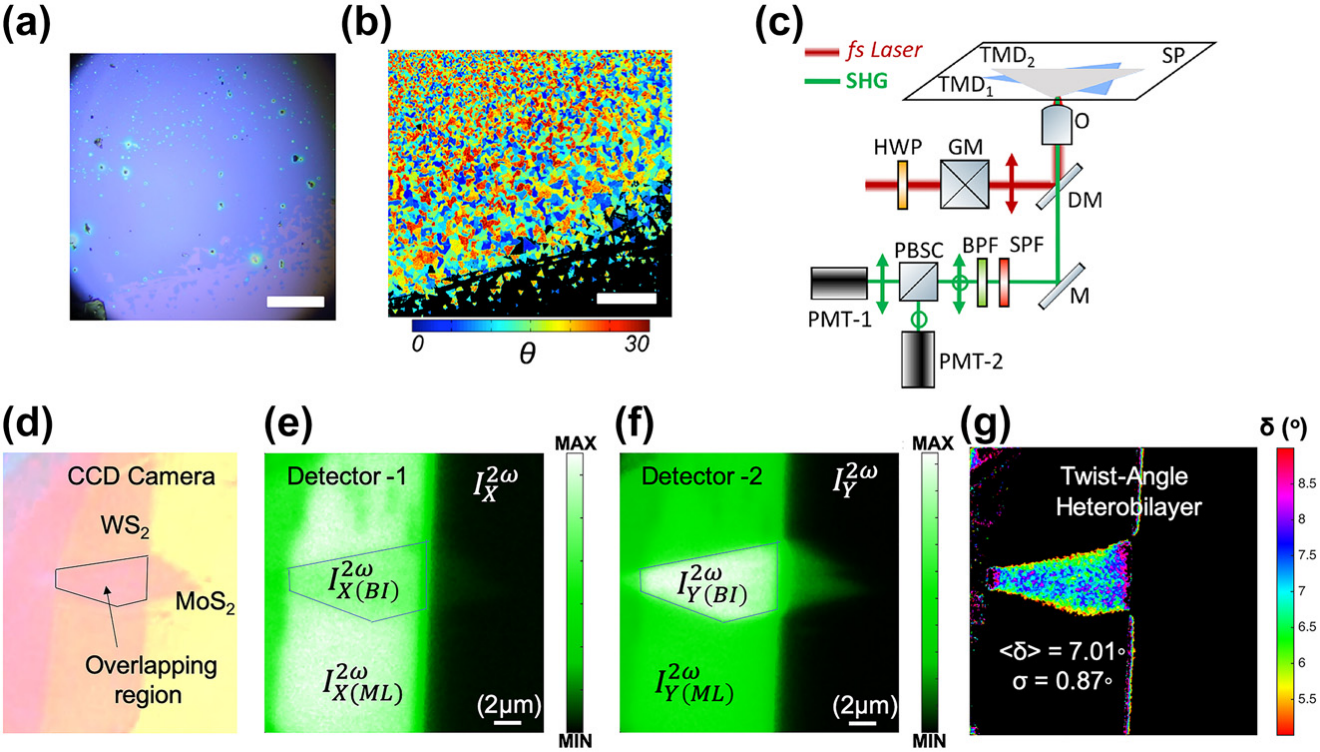
Single-scan extraction of twist-angle in 2D TMD heterobilayers. (a) Individual grains and as seen under a conventional microscope. (b) The color-mapped armchair direction image was produced by two SHG scans. Scale bar indicates 250 μm. (c) Illustration of the experimental configuration for single-scan calculation of twist-angle in 2D TMD heterobilayers. The linear polarization of the laser beam is parallel to *X*-axis (*φ* = 0°) and a polarization beam-splitter cube is placed in front of two detectors. Simultaneously, during a single-scan, the detector-1 records the $I_{X}^{2\omega }$ and the detector-2 records the $I_{Y}^{2\omega }$ component of the SHG. (d) CCD image of a WS_2_/MoS_2_ heterobilayer. The regions of WS_2_ monolayer, MoS_2_ monolayer, and their overlapping region are denoted. (e) SHG intensity image of the WS_2_/MoS_2_ heterobilayer, for the parallel to *X*-axis SHG component, $I_{X}^{2\omega }$ and (f) for the perpendicular component, $I_{Y}^{2\omega }$ . (g) The resulted twist-angle *δ* of the WS_2_/MoS_2_ heterobilayer, after application of Eq. (20), using a single-scan. (a) and (b) are reproduced with permission from [63]. Copyright 2015. AIP publishing. (c)–(g) are reproduced with permission from [81]. Copyright 2021. IOP Publishing Ltd.

It was demonstrated experimentally that *by* fixing the excitation linear polarization parallel to *X*-axis ( $\varphi =0^{◦}$ in Eq. (15)) and by performing two SHG intensity measurements ( $I_{X}^{2\omega }$ for parallel (*ζ* = 0°) and $I_{Y}^{2\omega }$ for perpendicular (*ζ* = 90°) to *X*-axis SHG detection), the armchair direction can be calculated in the range [0–30°] [63]. In (Figure 4(a)) [63] the application of Eq. (15) and the resulted mapping of the armchair crystal orientations for a big area is presented [63].

In the case of 2D TMD heterobilayers, in the overlapping region, the SHG signals of the two different TMD monolayers interfere and the overall produced SHG is governed by their vectorial addition. For polarizer *parallel* to *X*-axis (i.e. *ζ* = 0°), the SHG from *N* stacked TMD monolayers is described by (*φ* = 0°) [81]:

(16) $$I_{X}^{2\omega }={\left|\overset{N}{\underset{i=1}{\sum }}A_{i}\cos3\theta_{i}\right|}^{2}.$$

In the case of a hterobilayer (BI), Eq. (16) with *N* = 2 and *A*_1_ ≠ *A*_2_ reduces to:

(17) $$I_{X\left(BI\right)}^{2\omega }={\left[A_{1}cos3\theta_{1}+A_{2}cos3\theta_{2}\right]}^{2}.$$

If we now choose SHG detection *perpendicular* to *X*-axis (i.e. *ζ* = 90°), the SHG produced from the 2D heterostructure is given by:

(18) $$I_{Y\left(BI\right)}^{2\omega }={\left[A_{1}sin3\theta_{1}+A_{2}sin3\theta_{2}\right]}^{2}.$$

Then the unknown armchair direction *θ*_2_ is obtained using two SHG intensity measurements in the bilayer (BI) region (one for *ζ* = 0°, i.e. $I_{X\left(BI\right)}^{2\omega }$ and one for *ζ* = 90°, i.e. $I_{Y\left(BI\right)}^{2\omega }$ ) and two SHG intensity measurements in the monolayer (ML) region (one for *ζ* = 0°, i.e. $I_{X\left(ML\right)}^{2\omega }$ and one for *ζ* = 90°, i.e. $I_{Y\left(ML\right)}^{2\omega }$ ) as [81]:

(19) $$\theta_{2}=\frac{1}{3}tan^{-1}\frac{\sqrt{I_{Y\left(BI\right)}^{2\omega }}-\sqrt{I_{Y\left(ML\right)}^{2\omega }}}{\sqrt{I_{X\left(BI\right)}^{2\omega }}-\sqrt{I_{X\left(ML\right)}^{2\omega }}}.$$

If the measurements are performed simultaneously, e.g. by using a polarization beam splitter cube in front of two detectors (Figure 4(c)), the twist-angle *δ* can then be calculated with a single-scan using the formula [81]:

(20) $$\delta =\theta_{1}-\theta_{2}=\frac{1}{3}\left[tan^{-1}\sqrt{\frac{I_{Y\left(ML\right)}^{2\omega }}{I_{X\left(ML\right)}^{2\omega }}}-tan^{-1}\frac{\sqrt{I_{Y\left(BI\right)}^{2\omega }}-\sqrt{I_{Y\left(ML\right)}^{2\omega }}}{\sqrt{I_{X\left(BI\right)}^{2\omega }}-\sqrt{I_{X\left(ML\right)}^{2\omega }}}\right].$$

Experimental, calculation and mapping using a single-scan of the twist-angle in a WS_2_/MoS_2_ heterobilayer is presented in (Figure 4(g)) [81].

### 2.5 Polarization-resolved SHG in 2D metal monochalcogenides

**Figure 5:**
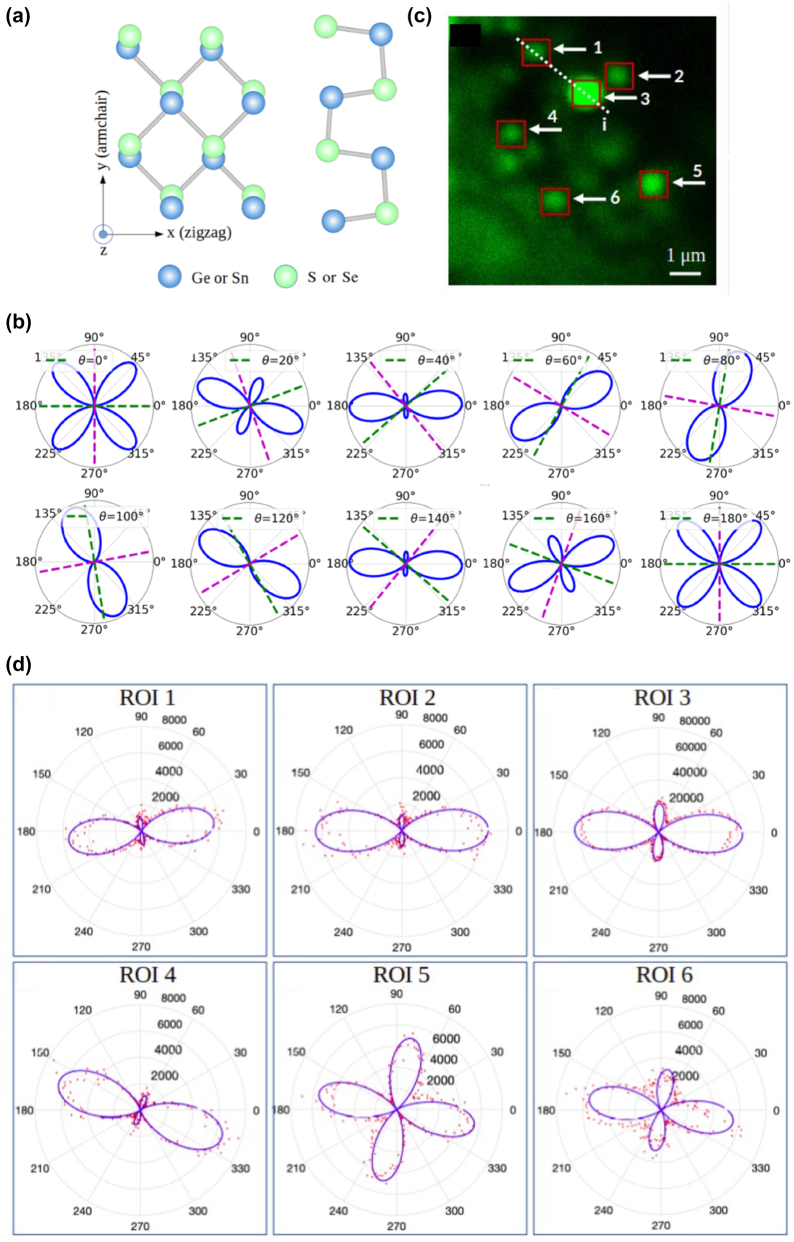
In-plane anisotropic P-SHG in 2D metal monochalcogenides. (a) Schematic of the crystal structure of orthorhombic 2D MXs, as seen from the top (left) and the side (right). (c) SHG imaging of 2D SnS flakes. (b) Numerical simulations of the theoretical P-SHG intensity produced by a 2D MX, described by Eq. (23). (d) Experimentally retrieved polar diagrams of the P-SHG intensity taken inside each ROI are depicted in (c). By fitting with Eq. (26) the armchair and the zig-zag crystallographic directions as well as the tensor element parameters *b* and *e*, for each ROI could be calculated [67]. The changes in the polar-diagrams for different flakes, is the signature of differences in their in-plane anisotropy. Figure is reproduced with permission from [67]. Copyright 2022. Wiley VCH.

The 2D Mχs are characterized by broken inversion symmetry (Figure 5(a)), a fact that renders them suitable for SHG conversion [82]–[85]. Indeed, using first-principles electronic structure theory, Wang and Qian theoretically predicted giant optical SHG in monolayer MXs [83]. They predicted that the strength of SHG susceptibility of GeSe and SnSe monolayers is more than one order of magnitude higher than that of monolayer MoS_2_. These results were also supported by another theoretical work by Panday and Fregoso [84]. Recently, Higashitarumizu et al. performed polarized SHG spectroscopy on a micrometer-size monolayer SnS [82], while Zhu et al. and Maragkakis et al. reported anisotropic SHG in few-layer SnS [67], [85].

The 2D MXs belong to the non-centrosymmetric, orthorhombic point group C_2v_ (mm^2^) [67], [83]. Thus, they have five independent, nonzero SHG susceptibility tensor elements, namely:

$$\chi_{\mathrm{yxx}}^{\left(2\right)},\chi_{\mathrm{yyy}}^{\left(2\right)},\chi_{\mathrm{yzz}}^{\left(2\right)}\;\text{and}\;\chi_{\mathrm{xyx}}^{\left(2\right)}=\chi_{\mathrm{xxy}}^{\left(2\right)}\;\text{and}\;\chi_{\mathrm{zzy}}^{\left(2\right)}=\chi_{\mathrm{zyz}}^{\left(2\right)}.$$

As a result, the nonlinear polarization can be written in matrix form as [67]:

(21) $$\begin{array}{ll} \left(\begin{array}{c} P_{x}^{2\omega }\\ P_{y}^{2\omega }\\ P_{z}^{2\omega } \end{array}\right) & =\varepsilon_{0}\left(\begin{array}{cccccc} 0 & 0 & 0 & 0 & 0 & \chi_{\mathrm{xxy}}^{\left(2\right)}\\ \chi_{\mathrm{yxx}}^{\left(2\right)} & \chi_{\mathrm{yyy}}^{\left(2\right)} & \chi_{\mathrm{yzz}}^{\left(2\right)} & 0 & 0 & 0\\ 0 & 0 & 0 & \chi_{\mathrm{zyz}}^{\left(2\right)} & 0 & 0 \end{array}\right)\left(\begin{array}{c} E_{x}^{\omega }E_{x}^{\omega }\\ E_{y}^{\omega }E_{y}^{\omega }\\ E_{z}^{\omega }E_{z}^{\omega }\\ 2E_{y}^{\omega }E_{z}^{\omega }\\ 2E_{x}^{\omega }E_{z}^{\omega }\\ 2E_{x}^{\omega }E_{y}^{\omega } \end{array}\right). \end{array}$$

Given that the excitation field is polarized along the sample plane, i.e. $E_{z}^{\omega }=0$ , the SHG equation is reduced to:

(22) $$\left(\begin{array}{c} P_{x}^{2\omega }\\ P_{y}^{2\omega } \end{array}\right)=\varepsilon_{0}E_{0}^{2}\left(\begin{array}{c} \chi_{\mathrm{xxy}}^{\left(2\right)}\sin\left[2\left(\varphi -\theta \right)\right]\\ \chi_{\mathrm{yxx}}^{\left(2\right)}cos^{2}\left(\varphi -\theta \right)+\chi_{\mathrm{yyy}}^{\left(2\right)}sin^{2}\left(\varphi -\theta \right) \end{array}\right).$$

This expression back to laboratory coordinates and for fixed polarizer before the detector at *ζ* = 0°, results in [67]:

(23) $$\begin{array}{ll} I_{X}^{2\omega } & ∼\frac{1}{16}\left[2\left(\chi_{\mathrm{yxx}}^{\left(2\right)}+\chi_{\mathrm{yyy}}^{\left(2\right)}\right)\sin\theta +\left(2\chi_{\mathrm{xxy}}^{\left(2\right)}-\chi_{\mathrm{yxx}}^{\left(2\right)}+\chi_{\mathrm{yyy}}^{\left(2\right)}\right)\right.\\ & \;\times \sin\left(\theta -2\varphi \right)+\left(2\chi_{\mathrm{xxy}}^{\left(2\right)}+\chi_{\mathrm{yxx}}^{\left(2\right)}-\chi_{\mathrm{yyy}}^{\left(2\right)}\right)\\ & \;{\left.\times \sin\left(3\theta -2\varphi \right)\right]}^{2}. \end{array}$$

The above Eq. (23) can be expressed in terms of dimensionless *χ* ^(2)^ tensor elements ratios, obtaining [67]:

(24) $$\begin{array}{ll} I_{X}^{2\omega } & =a\left[2\left(b+1\right)\sin\theta +\left(2c-b+1\right)\sin\left(\theta -2\varphi \right)\right.\\ & \;{\left.\times +\left(2c+b-1\right)\sin\left(3\theta -2\varphi \right)\right]}^{2}, \end{array}$$

where

(25) $$b=\chi_{\mathrm{yxx}}^{\left(2\right)}/\chi_{\mathrm{yyy}}^{\left(2\right)},c=\chi_{\mathrm{xxy}}^{\left(2\right)}/\chi_{\mathrm{yyy}}^{\left(2\right)},\;\text{and}\;a=\varepsilon_{0}^{2}E_{0}^{4}/\left[16{\left(\chi_{\mathrm{yyy}}^{\left(2\right)}\right)}^{2}\right]$$

is a multiplication factor. The SHG intensity can also be expressed in the equivalent form, which is used to fit the P-SHG experimental data [67]:

(26) $$\begin{array}{ll} I_{X}^{2\omega } & =a\left[2\left(b+1\right)\sin\theta +\left(b-1\right)\right.\\ & \;{\left.\left[\left(e-1\right)\sin\left(\theta -2\varphi \right)+\left(e+1\right)\sin\left(3\theta -2\varphi \right)\right]\right]}^{2}, \end{array}$$

where

(27) $$e=2c/\left(b-1\right).$$

In Figure 5(b), is presented the numerical simulation of the $I_{X}^{2\omega }$ modulation, described by Eq. (24), in polar plots, as a function of the orientation of the linear polarization of the excitation field, *φ*, for fixed values of *b*, *c*, and for different armchair directions, [67]. Three possible shapes are obtained: one with four symmetric lobes, one with four lobes symmetric in pairs, and one with two symmetric lobes.

The changes in the shape of the P-SHG polar diagrams, shown in Figure 5(b), reflect the in-plane anisotropy of the orthorhombic Mχs. The origin of this shape change is described by Eq. (24), where the SHG intensity depends on four parameters, i.e., *a*, *b*, *c,* and *θ*. It is therefore established a direct link between the P-SHG intensity modulation and the in-plane anisotropy of orthorhombic MXs through these four parameters. In particular, the shape of the theoretically predicted P-SHG polar diagrams shown in Figure 5(b) is determined by the corresponding armchair direction *θ* and the tensor elements ratios *b* and *c*. The parameters *b* and *c* denote the relative contribution of different directions to the SHG signals. Experimental demonstration of the effect of the in-plane anisotropy of orthorhombic SnS in the P-SHG measurements is presented in Figure 5(b) and (c) [67].

In contrast to the monolayer TMDs where the armchair direction can be calculated modulo 60° (due to their threefold rotational symmetry i.e., the fact that they have three equivalent armchair axes), in the case of 2D MXs, the armchair direction is unique. This is readily reflected in the SHG polar diagrams of MXs which are the same every 180° in the armchair direction (i.e. modulo 180°) [67].

### 2.6 Layer dependent SHG in 2D metal monochalcogenides

To describe the SHG intensity generated from a noncentrosymmetric orthorhombic 2D MX, with *N* number of layers, the interference model introduced for 2D TMDs [66], [79] is used. For example, SnS has been reported to exhibit two possible stacking sequences, the non-centrosymmetric AA and the centrosymmetric AB staking [82]. Experimental evidence has been provided that all ultrathin SnS flakes below a critical thickness, including the even-number layers, show SHG signals [82]. In any case, neglecting propagation effects, from an ultrathin noncentrosymmetric MX crystal which produces optical SHG, the second harmonic field arising will have the form of vector superposition:

(28) $$E^{2\omega }=E_{1}^{2\omega }+E_{2}^{2\omega }+\ldots +E_{N}^{2\omega },$$

where the indices denote the second harmonic signal from the corresponding layers. The total SHG intensity produced by the N-layer structure, will then be [67]:

(29) $$\begin{array}{ll} I_{N}^{2\omega } & ={\left|E_{1}^{2\omega }\right|}^{2}+{\left|E_{2}^{2\omega }\right|}^{2}+\ldots +{\left|E_{N}^{2\omega }\right|}^{2}\\ & \;+2E_{1}^{2\omega }\cdot E_{2}^{2\omega }+\ldots +2E_{N-1}^{2\omega }\cdot E_{N}^{2\omega } \end{array}$$

(30) $$\begin{array}{ll} I_{N}^{2\omega } & =I_{1}^{2\omega }+I_{2}^{2\omega }+\ldots +I_{N}^{2\omega }+2\sqrt{I_{1}^{2\omega }I_{2}^{2\omega }}\cos\delta_{1,2}+\ldots \\ & \;+2\sqrt{I_{N-1}^{2\omega }I_{N}^{2\omega }}\cos\delta_{N-1,N} \end{array}$$

where *δ*_*i*,*j*_, *i*, *j* = 1, 2, …, *N* denote the relative angle between layers *i* and *j*, i.e., the twist-angles. If for simplicity is assumed that the SHG intensity from the individual layers is equal ( $I_{1}^{2\omega }=I_{2}^{2\omega }=\ldots =I_{N}^{2\omega }=I_{\left(ML\right)}^{2\omega }$ ) and that the three layers are aligned (i.e., all twist-angles are zero), is found that [67]:

(31) $$I_{N}^{2\omega }=NI_{\left(ML\right)}^{2\omega }+I_{\left(ML\right)}^{2\omega }N\left(N-1\right)$$

(32) $$I_{N}^{2\omega }=N^{2}I_{\left(ML\right)}^{2\omega }.$$

This is the same with the previous result for TMD flakes with zero twist-angle, that the SHG intensity scales quadratically with the number of layers.

Polarized SHG has been widely used to characterize the crystal orientation for odd layers of 2H phases. While for other crystal forms of 2D materials belonging to different space groups, two-fold or four-fold pattern are employed as indicators of crystal orientation [86].

### 2.7 Polarization-resolved SHG probes valley polarization

Traditional optoelectronics relies on using light to control the movement of electronic charge, facilitating tasks such as information transmission, storage, and retrieval. In electronic setups featuring degenerate minima in their band structures – known as valleys – a supplementary parameter, the valley index, can function as a distinct carrier of information, marking these minima. This concept has paved the way for a novel domain in electronics called valleytronics. Valleytronics offers the potential to process extra information within the confines of the same physical area [87]–[89].

The underlying physical principle is the population imbalance between different valleys. This is reasonable considering that polarization and transport effects are associated with charge separation and local variations in the chemical potential, respectively. Hence, they directly reflect the crystal symmetries in both real and momentum space. In real space, the charge is locally accumulated around the atomic positions, whereas in momentum space, the carriers occupy states in the vicinity of high symmetry points within the hexagonal Brillouin zone [68]. Both polarization and transport effects are studied by analyzing the second-order nonlinear optical response of atomically thin crystals [68], [69], [90], [91].

**Figure 6:**
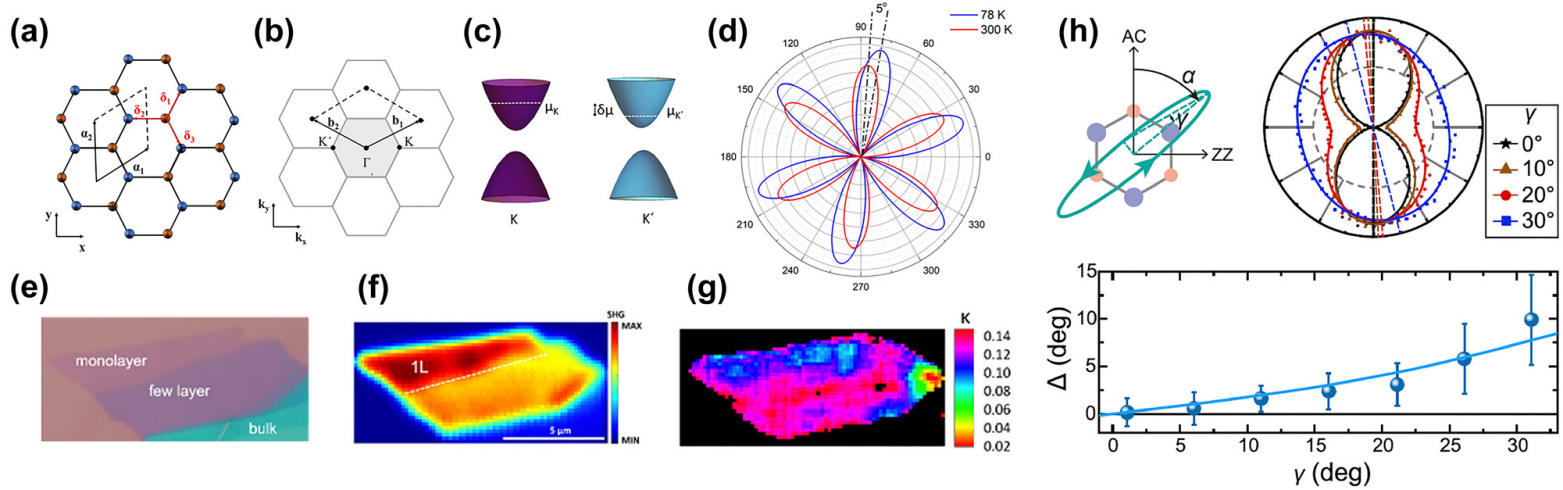
P-SHG probes valley polarization in 2D TMDs. (a) Top view of monolayer TMD with transition metal atoms in red and chalcogen atoms in blue. The lattice vectors *a*_1_ and *a*_2_ and first-neighbor vectors *δ*_1_, *δ*_2_, and *δ*_3_ are also shown. The region bounded by dashed lines corresponds to the primitive cell. (b) First Brillouin zone (shaded) of monolayer TMD showing the high symmetry points Γ, *K*, and *K*′. The reciprocal lattice vectors *b*_1_ and *b*_2_ are also depicted. (c) Schematic of monolayer TMD dispersion. The paraboloids represent valence and conduction bands at *K* (purple) and *K*′ (cyan) points. The white dashed lines indicate the chemical potential at different valleys for the case of population imbalance. (d) Experimental demonstration of temperature dependence of the P-SHG polar diagram due to valley population imbalance. With decreasing temperature, the SHG intensity increases due to the valley-induced contribution. A rotation of the polar diagram away from the main crystallographic axis is clearly seen [68]. (e) Optical image of a WS_2_ flake consisting of monolayer, few layer and bulk regions. (f) SHG intensity mapping of the WS_2_ flake at 78 K. (g) Mapping of valley population imbalance in terms of the dimensionless parameter *κ* at 78 K. (h) Measurement of valley polarization at room temperature by changing the ellipticity *γ*(°) of the excitation field and measuring the rotation Δ(°) of the produced SHG elliptical polar diagram. (a)–(g) are reproduced with permission from [68]. Copyright 2021. Springer Nature. (h) is reproduced with permission from [69]. Copyright 2020. American Chemical Society.

Recent studies suggest that the second-order optical response is a useful tool to probe the electronic configuration of 2D crystals [90], [92], [93]. This is possible due to the symmetry characterizing the hexagonal Brillouin zone in momentum space. Similarly, to the alternating atoms at the corners of the hexagon in real space, characterized by the D_3h_ symmetry (Figure 6(a)), in momentum space the alternating *K* and *K*′ points also result in D_3h_ symmetry, reflecting the trigonal warping of electrons in the vicinity of high symmetry points (Figure 6(b)) [68]. A direct consequence is the capability for such crystals to produce valley-induced SHG, additionally to the intrinsic second-order response. As a result, in the presence of population imbalance between the two valleys additional elements in the second-order nonlinear optical susceptibility tensor become nonzero [68], [94]:

$$\chi_{\mathrm{yyy}}^{\left(2\right)}=-\chi_{\mathrm{yxx}}^{\left(2\right)}=-\chi_{\mathrm{xyx}}^{\left(2\right)}=\chi_{\text{vpi}}^{\left(2\right)},$$

where $\chi_{\text{vpi}}^{\left(2\right)}$ is the nonzero element of the valley population imbalance (VPI)-induced second order susceptibility tensor.

Notably, since the VPI defines the difference between the valley populations,

(33) $$\delta n=n_{K}-n_{k},$$

it also reflects the chemical potential difference, *δμ*, between the two valleys (Figure 6(c)). Hence, in the presence of imbalance, the additional valley-induced contribution to the SHG that is intrinsically generated by the TMD crystal, can be estimated as [68]

(34) $$I_{\text{SHG}}^{\text{VPI}}∼\delta n^{2}∼\delta \mu^{2}$$

with the corresponding contribution to the second-order nonlinear susceptibility being proportional to the chemical potential difference, *δμ* [68]. Accordingly, local variations in the chemical potential affect the SHG induced by the VPI and thus can be probed by nonlinear optical experiments [68]. More importantly, and in contrast to the intrinsic nonlinear optical response of 2D TMDs, the valley-induced SHG is sensitive to temperature variations. Based on this, by varying the temperature of a 2D TMD crystal and the corresponding changes in the SHG intensity are used to probe the intervalley chemical potential difference and therefore the VPI [68].

An experimental SHG measurement of VPI was first proposed by Hipolito and Pereira [90], in which a quarter-wave plate is placed before the sample with its fast axis at an angle *α*. Experimentally the above concept was utilized by Mouchliadis et al. [68] using a half-wave plate to control the orientation *φ* of the fundamental linear polarization, while a linear polarizer placed before the detector at an angle *ζ*, selects suitable SHG components. The linear polarization of the excitation changes to elliptical as the beam passes through the quarter-wave plate whose axis has an offset of 5° with respect to the polarization axis. Such a configuration provides the means for an asymmetric population of the two valleys [68], [69], [91].

Application of nonlinear optics for a crystal with D_3h_ symmetry in the presence of VPI yields the SHG field emerging from the crystal as [68]:

(35) $$\left(\begin{array}{c} P_{x}^{2\omega }\\ P_{y}^{2\omega } \end{array}\right)∼\left(\begin{array}{c} \chi_{\text{int}}^{\left(2\right)}\left[{\left(E_{x}^{\omega }\right)}^{2}-{\left(E_{y}^{\omega }\right)}^{2}\right]-2\chi_{\text{vpi}}^{\left(2\right)}E_{x}^{\omega }E_{y}^{\omega }\\ -\chi_{\text{vpi}}^{\left(2\right)}\left[{\left(E_{x}^{\omega }\right)}^{2}-{\left(E_{y}^{\omega }\right)}^{2}\right]-2\chi_{\text{int}}^{\left(2\right)}E_{x}^{\omega }E_{y}^{\omega } \end{array}\right).$$

Here $\chi_{\text{int}}^{\left(2\right)}$ and $\chi_{\text{vpi}}^{\left(2\right)}$ correspond to the intrinsic and induced due to VPI contributions to the second-order response, respectively.

This means that the SHG intensity reaching the detector depends on four angles, namely *φ*, *θ*, *α*, *ζ*, corresponding to the effects of excitation linear polarization, crystal orientation, quarter-wave plate, and analyzer, respectively. In this case, the detected SHG intensity is given by [68]:

(36) $$\begin{array}{c} I_{\text{vpi}}^{2\omega }=A{\left[\cos\left(2\alpha +\zeta -3\theta \right)-\kappa \sin\left(2a+\zeta -3\theta \right)\right]}^{2}\\ +sin^{2}2\left(\alpha -\varphi \right){\left[\kappa \cos\left(2a+\zeta -3\theta \right)+\sin\left(2a+\zeta -3\theta \right)\right]}^{2} \end{array}$$

where *κ* denotes the magnitude of VPI-induced to intrinsic susceptibility ratio [68], [69], [91],

(37) $$\kappa =\frac{\left|\chi_{\text{vpi}}^{\left(2\right)}\right|}{\left|\chi_{\text{int}}^{\left(2\right)}\right|}$$

and *A* is a multiplication factor that depends on the amplitude of the field and the intrinsic second-order susceptibility.

The ratio *κ* which indicates the contribution of VPI to the P-SHG modulation can be extracted upon fitting the experimentally measured P-SHG intensity with Eq. (36) [68]. In this experimental configuration, *φ* = *ζ* that gives rise to a six-petal pattern for the P-SHG intensity (Figure 6(d)). Temperature dependent mapping of *κ* parameter in monolayer, few layer and bulk of exfoliated WS_2_ crystal has been presented by Mouchliadis et al. [68]. In another experimental configuration the degree of valley polarization is calculated at room temperature by changing the ellipticity *γ*(°) of the excitation field (thus exciting with elliptical polarization) and measuring the rotation (Δ(°) = $tan^{-1}\frac{\chi_{\text{vpi}}^{\left(2\right)}}{\chi_{\text{int}}^{\left(2\right)}}$ ) of the produced elliptical SHG polar diagram. This is done by rotating a linear polarizer before the detector (Figure 6(h)) [69], [91].

### 2.8 Polarization-resolved SHG maps strain

The properties of 2D materials can be strongly influenced by strain making them promising candidates for stretchable and flexible electronics [95]. For flexible device design using 2D materials, it is of significant importance to engineer their properties in a controllable way. Currently, strain engineering represents a very efficient and powerful route for this purpose [95], [96], benefiting from the flexibility of 2D materials. Accordingly, the development of suitable techniques to precisely characterize both the amplitude and direction of the local strain vector is a prerequisite for future applications [70], [71].

Thus, it is essential to figure out the strain tensor distribution in 2D materials. The direct impact of st_r_ain on 2D materials is lattice deformation and symmetry variations. As we saw above, the second-order nonlinear susceptibility $\chi_{\text{int}}^{\left(2\right)}$ is sensitive to crystallographic direction, therefore strain will change the lattice structure and thus the optical susceptibility of the material. The second order nonlinear susceptibility after the application of strain reads [70], [71]:

$$\chi_{st}^{\left(2\right)}=\chi_{\text{int}}^{\left(2\right)}+p_{\mathrm{ijklm}}u_{lm}$$

where *p*_*ijklm*_ is the photoelastic tensor and *u*_*lm*_ is the strain tensor. The $\chi_{\text{int}}^{\left(2\right)}$ describes the second-order nonlinear susceptibility of the unstrained crystal. The parameters of *p*_*ijklm*_ and $\chi_{st}^{\left(2\right)}$ can be obtained by applying different levels of uniaxial strain combined with P-SHG signals, and finally the strain tensor is accessed [70], [71].

For TMD monolayer, in the P- SHG measurements the linear polarized incident electric field is under angle *φ* and the SHG signal is analyzed with same polarization, i.e. *ζ* = *φ*. The SHG under strain is then given by [71]:

(38) $$I_{\zeta =\varphi }^{2\omega }∼\frac{1}{4}{\left[Acos3\varphi +B\cos\left(2\theta +\varphi \right)\right]}^{2}$$

where,

(39) $$A=\left(1-v\right)\left(p_{1}+p_{2}\right)\left(\varepsilon_{xx}+\varepsilon_{yy}\right)+2\chi_{\text{int}}^{\left(2\right)}$$

and

(40) $$B=\left(1+v\right)\left(p_{1}-p_{2}\right)\left(\varepsilon_{xx}-\varepsilon_{yy}\right)+2\chi_{\text{int}}^{\left(2\right)}$$

The *p*_1_ and *p*_2_ are the photoelastic parameters, *ɛ*_*xx*_ and *ɛ*_*yy*_ denote the principal strains, *θ* is the principal strain orientation and *χ*_int_ the nonlinear susceptibility parameter of the unstrained crystal lattice.

**Figure 7:**
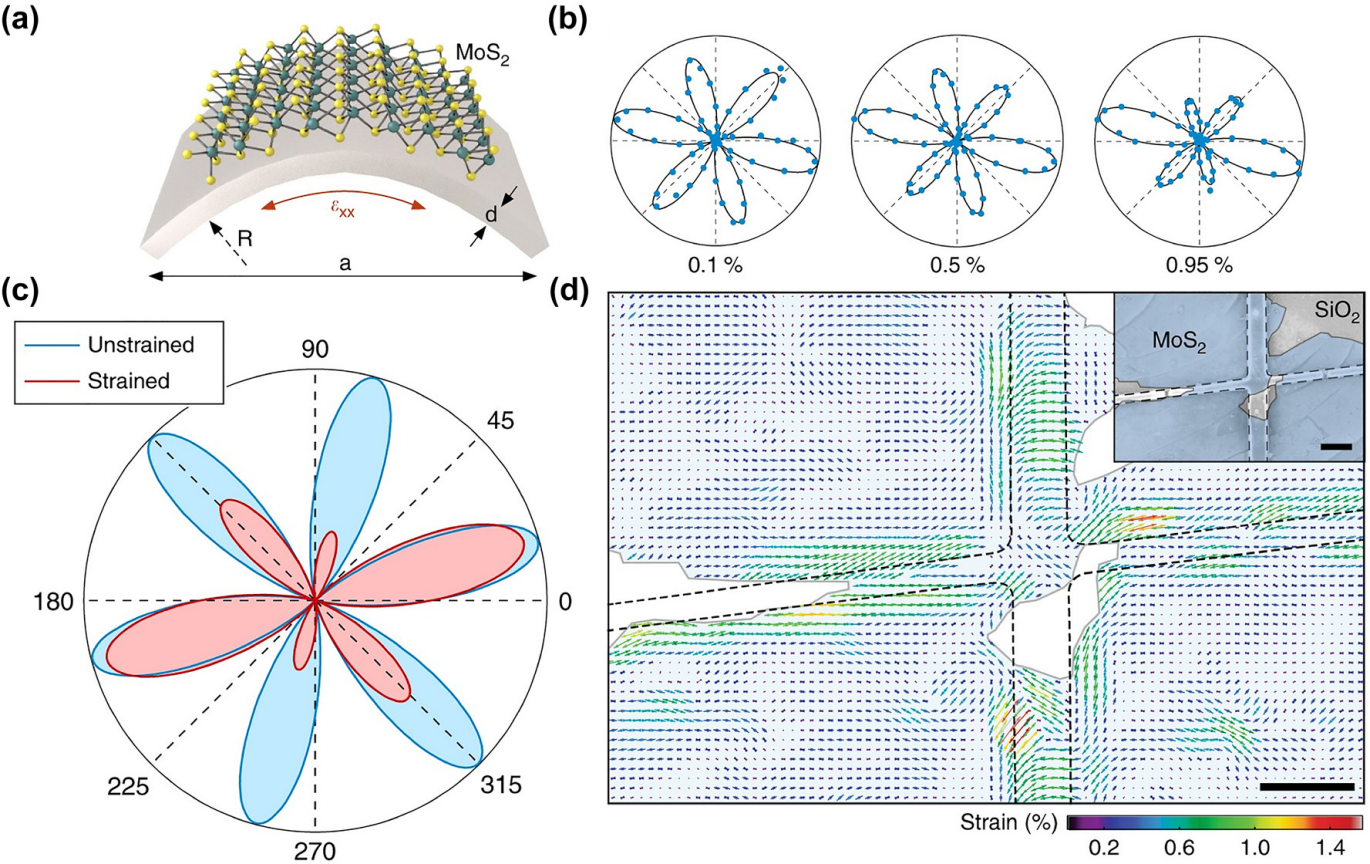
Mapping strain using P-SHG. (a) Schematic illustration of the experimental bending method in a 2D MoS_2_ monolayer. (b) Experimentally retrieved P-SHG polar plots for applied tensile strains of 0.1 %, 0.5 %, and 0.95 % and fitting with Eq. (38). (c) P-SHG polar plot showing unstrained TMD crystal (blue) and P-SHG polar plot from a TMD crystal with 1.0 % tensile strain in horizontal (0°) direction. (d) Pixel wise strain map of a MoS_2_ monolayer (filled color) on a lithographically defined structure (dashed lines). Arrows show the result of the fitting and the calculated uniaxial strain. Inset: SEM image of the sample. Scale bars, 1 μm. Figure is reproduced with permission from [71]. Copyright 2018. Springer Nature.

The applied uniaxial tensile strain (Figure 7(a)) alters the shape and intensity of P-SHG patterns (Figure 7(b) and (c)) from which parameters in the photoelastic tensor as well as the strain tensor can be extracted by fitting Eq. (38). Based on this, it is convenient to visualize the strain field of a certain area combined with the P-SHG mapping (Figure 7(d)) [71]. The inset of Figure 7(d) shows the scanning electron microscopy (SEM) image of the MoS_2_ monolayer lying on a lithographically defined structure. The MoS_2_ membrane undergoes inhomogeneous strain tensor distribution across the grids. Figure 7(d) shows the strain mapping by arrows after the pixel-by-pixel fitting of the P-SHG experimental data to Eq. (38).

## 3 Second harmonic generation in 2D perovskites

Halide perovskites are generally defined as a network of corner-sharing [BX_6_]^4−^ octahedra that crystallize with a general ABX_3_ (or equivalent) stoichiometry, where A and B denote cations, and X represent halide anions [97]. They have attracted significant attention from the scientific community due to their superior optical properties, ability to tune bandgaps by varying the composition, and ease of synthesis methods. Recently, in addition to 3D perovskites, layered 2D perovskites have also started to attract research attention. From a purist’s perspective, the notion of a 2D perovskite presents an inherent paradox. The very definition of a perovskite structure necessitates a three-dimensional framework of corner-sharing [BX_6_]^4−^ octahedra extending infinitely [97]. However, the terminology “2D perovskite” has become so ubiquitous in the scientific community that we shall adhere to this convention for the purposes of this review. The advantages of 2D materials compared to 3D analogues are that they have more ability to tune properties by widely changing the functional organic cations or increasing the thickness of the perovskite layer [98]. Their unique structure, leading to strong quantum confinement and excitonic effects, can lead to significant enhancement of nonlinear optical properties (and second harmonic generation in particular) compared to their 3D counterparts [99], [100].

Therefore, 2D organic–inorganic hybrid perovskites are promising materials with many potential applications in light-emitting, sensing and other optical devices. In this review we will focus on their application for SHG.

### 3.1 General properties of 2D perovskites

**Figure 8:**
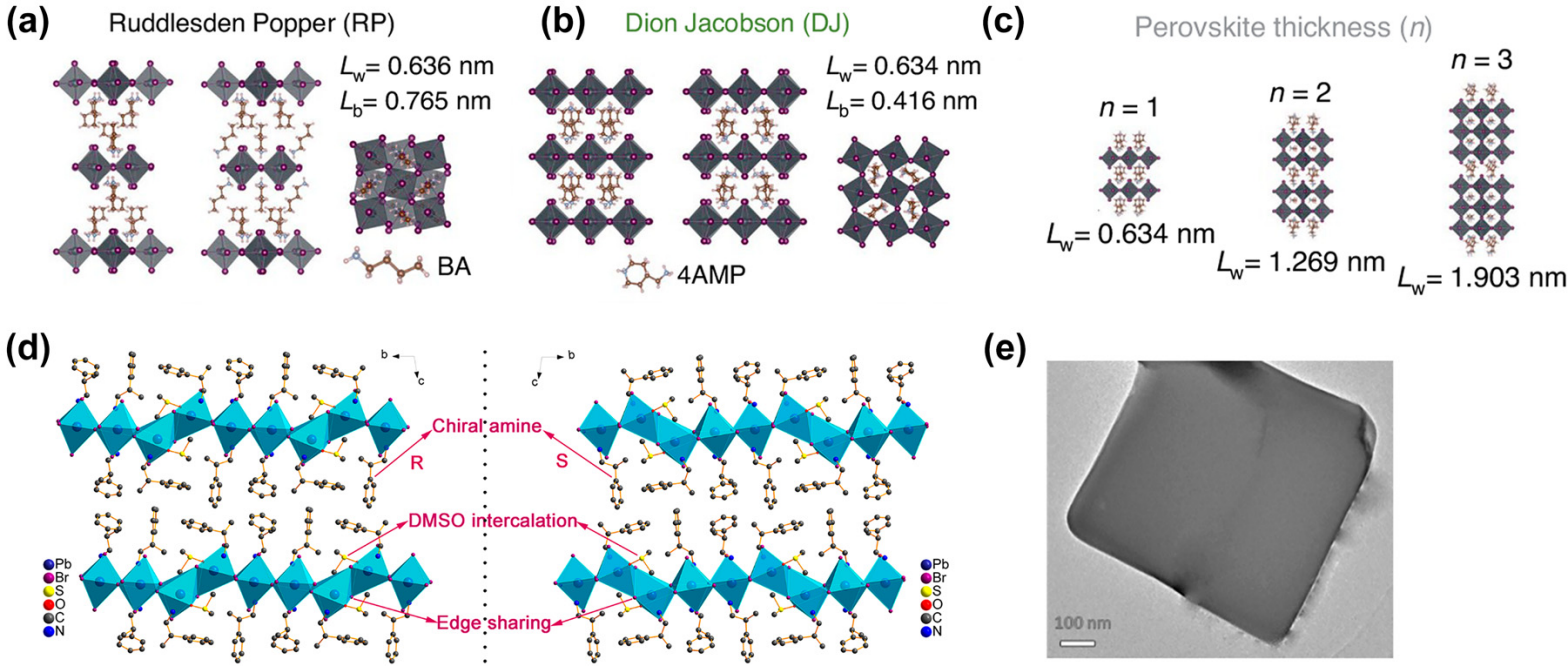
Structure of 2D perovskites. (a), (b) Example structures of R-P and D-J perovskite phases, BA – butylammonium; 4AMP – 4-(aminomethyl) piperidinium. (c) Example of RP crystal structures with different perovskite thicknesses defined by the number *n* (number of octahedra along the stacking direction in the perovskite layer). (d) Crystallographic structure of the chiral perovskites (R-β-methylphenethylamine)_1.5_PbBr_3.5_(DMSO)_0.5_ and (S-β-methylphenethylamine)_1.5_PbBr_3.5_(DMSO)_0.5_, respectively. (e) TEM bright-field image of [(phenylmethylammonium)_2_]PbCl_4_ nanosheet. (a), (b), (c) are reproduced from [104] with permission from Springer Nature. (d) is reprinted with permission from [103]. Copyright 2018, American Chemical Society. (e) is reprinted with permission from [105]. Copyright 2019, American Chemical Society.

Generally, 2D layered perovskites are multiple quantum well (MQW) structures, where a 2D layer of [BX_6_]^4−^ octahedra functions as a quantum well and layers of bulk organic anions act as a barrier. In the chemistry of layered perovskites, two main phases are defined in the following way: the Dion–Jacobson (DJ) phases A′A_*n*−1_B_*n*_X_3*n*+1_ (Figure 8(b)) have one interlayer cation A′ per formula unit, while the Ruddlesden–Popper (RP) phases A′_2_A_*n*−1_B_*n*_X_3*n*+1_ (Figure 8(a)) have two interlayer cations A′ per formula unit. The main structural feature of RP and DJ inorganic layered perovskites is the displacement of adjacent perovskite layers in (*a*, *b*) layer plane. In RP phases, a shift (*a* + *b*)/2 is observed (where *a* and *b* are in-plane parameters corresponding to the length of one octahedron) as a result of the capped square anti-prismatic coordination of cations in the interlayer space. In DJ phases, displacement of neighboring perovskite layers relative to each other in the plane of layers (*a*, *b*) does not occur, since the interlayer cations adopt cubic type coordination [101]. Additionally, ACI (alternating cation in the interlayer space) phase of 2D perovskites is often mentioned [102], which consists of two different alternating cations in the interlayer space. In this work we also consider perovskite-like structures, which cannot be attributed to the above phases (for example, R/S-(MPEA)_1.5_PbBr_3.5_(DMSO)_0.5_ [103], Figure 8(d)), but have the main features of 2D perovskites.

Table 2 presents chemical composition and strength of second order nonlinear effects for 2D perovskites and perovskite-like structures, described in the literature.

**Table 2:** Reported 2D perovskite materials for SHG.

Chemical formula	Phase	*χ* ^(2)^, pm/V	$\frac{\chi^{\left(2\right)}}{\chi_{\mathrm{KDP}}^{\left(2\right)}}$	*λ*_exc._, nm	Ref.
(n-butylammonium)_2_CsPb_2_Br_7_	R-P	0.62	0.70	1,064	[106]
[n-butylammonium]_2_(methylammonium)Pb_2_Br_7_	R-P	0.56	0.64	1,064	[107]
(n-butylammonium)_2_(ethylammonium)_2_Pb_3_I_10_	R-P	0.56	0.64	1,064	[108]
R/S-(β-methylphenethylamine)_1.5_PbBr_3.5_(DMSO)_0.5_	–	1.36	1.55	850	[103]
[R/S-1-(4-chlorophenyl)ethylammonium]_2_PbI_4_	–	0.62	0.70	1,064	[109]
(isoamylammonium)_2_(ethylammonium)_2_Pb_3_Br_10_	R-P	0.51	0.58	1,300	[110]
(*p*-bromobenzylammonium)_2_(ethylammonium)_2_Pb_3_Br_10_	R-P	0.92	1.05	1,340	[111]
[(R/S)- β-methylphenethylamine]_2_PbBr_4_	–	0.64	0.73	1,064	[112]
(N-methyl-iodoproylammonium)_2_PbI_4_	R-P	5.73	6.51	1,580	[113]
(3-(aminomethyl)pyridinium)GeI_4_	D-J	0.69	0.78	1,064	[114]
(4-(aminomethyl)pyridinium)GeI_4_	D-J	0.68	0.77	1,064	[114]
(3-(aminomethyl)pyridinium)(methylammonium,)Ge_2_I_7_	D-J	0.74	0.84	1,064	[114]
(4-(aminomethyl)pyridinium)(methylammonium,)Ge_2_I_7_	D-J	0.65	0.74	1,064	[114]
[2-fluorobenzylammonium]_2_PbCl_4_	D-J	0.83	0.94	1,064	[115]
(4-(aminomethyl)piperidinium)_2_AgBiBr_8_·H_2_O	–	0.88	1.00	1,064	[116]
(4-(aminomethyl)piperidinium)_2_AgBiBr_8_·0.5H_2_O	–	0.51	0.58	1,064	[116]
(n-butylammonium)_2_(formamidinium)Ge_2_I_7_	R-P	17.5	19.9	850 to 930	[117]
(n-butylammonium)_2_(formamidinium)Sn_2_I_7_	R-P	0.63	0.72	850 to 930	[117]
(n-butylammonium)_2_(formamidinium)Pb_2_I_7_	R-P	0.19	0.22	850 to 930	[117]
(n-butylammonium)_2_(methylammonium)Ge_2_I_7_	R-P	13.10	14.89	850 to 930	[117]
(n-butylammonium)_2_(methylammonium)Sn_2_I_7_	R-P	0.17	0.19	850 to 930	[117]
(n-butylammonium)_2_(methylammonium)Pb_2_I_7_	R-P	0.05	0.06	850 to 930	[117]
(methylhydrazinium)_2_PbBr_4_	R-P	0.28	0.32	1,300	[118]
(n-butylammonium)_2_(methylammonium)_*n*−1_Pb_n_I_3*n*+1_	R-P	0.067	0.08	1800	[119]
(R-/S-1-(4-chlorophenyl)ethylamine)_4_Bi_2_I_10_	–	3.80	4.32	2060	[120]
(R-1-(1-naphthyl)ethylammonium])_2_PbBr_4_	–	0.60	0.68	1,064	[121]
(4,4-difluorocyclohexylammonium)_2_PbBr_4_	–	1.14	1.3	1,064	[122]
(2-Methyl-1,5-diaminopentaneH_2_)PbBr_4_	R-P	–	–	–	[123]
[(phenylmethylammonium)_2_]PbCl_4_	R-P	–	–	–	[105]
(RNH_3_)_2_(methylammonium)_*n*−1_Pb_*n*_I_3*n*+1_, RNH_3_ = PEA (phenylethylammonium), TPMA (2-thiophenemethylammonium); *n* = 2,3	R-P	–	–	–	[124]
(4-Bromo-2-fluorobenzylammonium)_2_CsGe_2_I_7_	R-P	–	–	–	[125]
[Methylhydrazinium]_2_PbCl_4_	R-P	–	–	–	[126]

*χ* ^(2)^ of materials calculated from the following expression:

(41) $$\chi_{S}^{\left(2\right)}=\chi_{R}^{\left(2\right)}{\left|\frac{I_{S}\left(2\omega \right)}{I_{R}\left(2\omega \right)}\right|}^{1/2}$$

During the calculations, the following values were used as reference [127]:

$$\begin{array}{ll} X_{\text{KDP}}^{\left(2\right)} & =0.88\;\text{pm/V}\left(1064\;\text{nm}\right);X_{\alpha -quartz}^{\left(2\right)}\\ & =0.67\;\text{pm/V}\left(1064\;\text{nm}\right) \end{array}$$

### 3.2 Formation of second order nonlinearity

It needs to be noted that the majority of 2D perovskites do not exhibit second order NLO due to the structure symmetry. Therefore, for SHG signal to be present centrosymmetry should be broken. Thus, here we discuss the influence of composition on the structural properties and symmetry breaking in 2D perovskites A′A_*n*−1_B_*n*_X_3*n*+1_/A′_2_A_*n*−1_B_n_X_3*n*+1_.

#### 3.2.1 A′-(spacer) cation influence

Although the optical properties of 2D perovskites are mostly dependent on the inorganic layer, the steric effect of the organic cation can induce distortion in the crystal lattice. One of the main ways to introduce second-order nonlinearities (and therefore SHG) in 2D perovskites is the use of chiral spacer A′-site cations, which can lead to the formation of non-centrosymmetric 2D perovskite structures due to intrinsic centrosymmetry breaking in chiral structures. Recently, several works have been published that raise this topic [103], [109], [112], [120]. However, enantiomeric pure chiral ammonium cations can be expensive due to problems associated with asymmetric synthesis and chiral resolution techniques [113].

**Figure 9:**
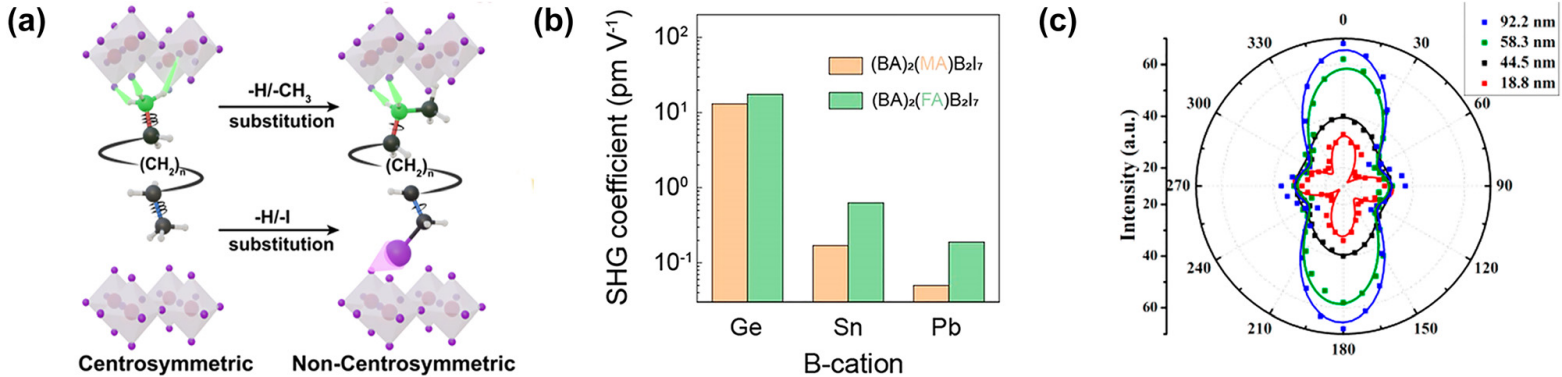
Tuning of SHG in 2D perovskites through composition. (a) Schematic showing the design of the organic A′-site cation used to obtain non-centrosymmetric hybrid 2D perovskites A_2_PbI_4_. (b) Comparison of the SHG coefficients in (BA)_2_(A)B_2_I_7_ with different A- and B-cations. (c) Polar SHG intensity plots for [(C_6_H_5_CH_2_NH_3_)_2_]PbCl_4_ nanosheets with different thickness. (a) is reprinted with permission from [113]. Copyright 2023, American Chemical Society. (b) is reprinted with permission from [117]. Copyright 2022, American Chemical Society. (c) is reprinted with permission from [105]. Copyright 2019, American Chemical Society.

Alternatively, R. Chakraborty et al., in an article devoted to the design of non-centrosymmetric hybrid halide perovskites, developed the approach for obtaining non-centrosymmetric structure by using achiral spacer cations through lowering the rotational symmetry at both ends of the spacer cation A′, which should lead to heterogeneous hydrogen-iodine and iodine-iodine interactions at the organic-inorganic interface of 2D perovskites, which break the local inversion symmetry of Pb-I octahedra [113]. In this work, the authors demonstrated the synthesis of a non-centrosymmetric 2D perovskite (MIPA)_2_PbI_4_ (where MIPA is N-methyl-iodopropylammonium), the break of the central symmetry of which arose due to the substitution of a –H (in –NH3^+^) with –CH_3_ group and substitution of a –H (in the hydrocarbon skeleton) with –I, leading to a significant change in hydrogen bond interactions (Figure 9(a), Table 3).

#### 3.2.2 A-cation influence

In case of 2D perovskites with number of layers *n* ≥ 2 in addition to interlayer spacer A′ cations, smaller different A-cations located between *n* layers of octahedra may also be present. Xinyu Li et al. studied the influence of the organic A cation on the structural properties of 2D perovskites (BA)_2_(A)Sn_2_I_7_ [117]. They demonstrated that as the size of cation A increases, the average Sn–I bond length and volume of the perovskite cage also generally increases, and elongation of the Sn–I bond length increases the tendency of B-cation displacement from the center. Displacement of the B cation breaks the central symmetry and allows second-order nonlinear optical effects (Figure 9(b), Table 3).

#### 3.2.3 B-cation influence

Varying B-cation in (BA)_2_(MA)B_2_I_7_ 2D perovskites, Xinyu Li et al. [117] demonstrated the increased lone-pair expression in following order of B cations: Pb^2+^ < Sn^2+^ < Ge^2+^, that has two origins. First, in the Pb^2+^ < Sn^2+^ < Ge^2+^ trend, the covalent interaction between the B cations and the p-orbitals of the halide anions increases as a result of the relativistic contraction of the ns^2^ orbital. Second, as the radius of the B-cation decreases, i.e., Pb^2+^ > Sn^2+^ > Ge^2+^, the methylammonium (MA) cation becomes too big for the corresponding metal halide framework; this expands the perovskite cage. As a result, increasing the B–I bond length increases the tendency of the B-cation off-center displacement (Figure 9(b), Table 3).

#### 3.2.4 X-anion influence

The size and polarizability of the X anion also have a major influence on the overall symmetry of the perovskite crystal lattice. Larger halogen anions distort the crystal lattice more than smaller ones, and this distortion can break central symmetry. Furthermore, recent findings [128] demonstrate that methylhydrazine’s nitrogen atoms can participate in coordination with lead atoms, alongside halides. This interaction activates a previously stereo-inactive electron pair, leading to significant alterations in the system’s symmetry.

**Table 3:** Influence of the composition and structure of 2D perovskites on second-order nonlinearity.

Tuning parameter	Material	Result	Figure	Ref.
A′-cation	(IPA)_2_PbI_4_ (MIPA)_2_PbI_4_	(IPA)_2_PbI_4_: centrosymmetric (MIPA)_2_PbI_4_: *χ* ^(2)^ = 5.73 pm/V	Figure 9(a)	[113]
A-cation	(BA)_2_(A)Ge_2_I_7_	(BA)_2_(MA)Ge_2_I_7_: *χ* ^(2)^ = 13.1 pm/V (BA)_2_(FA)Ge_2_I_7_: *χ* ^(2)^ = 17.5 pm/V	Figure 9(b)	[117]
B-cation	(BA)_2_(FA)B_2_I_7_	(BA)_2_(FA)Ge_2_I_7_: *χ* ^(2)^ = 17.5 pm/V; (BA)_2_(FA)Sn_2_I_7_: *χ* ^(2)^ = 0.63 pm/V; (BA)_2_(FA)Pb_2_I_7_: *χ* ^(2)^ = 0.19 pm/V	Figure 9(b)	[117]
Layer thickness	[(C_6_H_5_CH_2_NH_3_)_2_]PbCl_4_	d_33_/d_24_ ratios are, respectively, 1.46 and 1.17 for the 92.2 and 18.8 nm nanosheets	Figure 9(c)	[105]

#### 3.2.5 Layer thickness influence

The thickness of the multilayer structure formed by 2D perovskites also has a major influence on the resulting nonlinear optical properties. Wen-Juan Wei et al. studied the influence of van der Waals interactions on the SHG response of 2D perovskite [(C_6_H_5_CH_2_NH_3_)_2_]PbCl_4_ [105]. They found that the induced second-order polarization occurs predominantly due to cations (C_6_H_5_CH_2_NH_3_)^+^; and these organic amine cations form significantly reorganized conformations with decreasing nanosheet thickness due to weakening van der Waals interactions. Since the orientation of organic components at the interface determines their electrical properties and, in particular, dipolar susceptibility, the difference in resulting structure leads to dramatic changes in the SHG properties. Results, presented by Wen-Juan Wei et al. show that the SHG intensity of these nanosheets increases with increasing layer thickness, while their in-plane anisotropy shows the opposite trend (Figure 9(c), Table 3).

### 3.3 Tuning of SHG response

Tuning the SH signal is an important task for many nonlinear photonics applications. Control of a nonlinear optical signal can be achieved by many different methods, including the use of electrical, thermal, optical, or other signals.

#### 3.3.1 Temperature-induced switching

**Figure 10:**
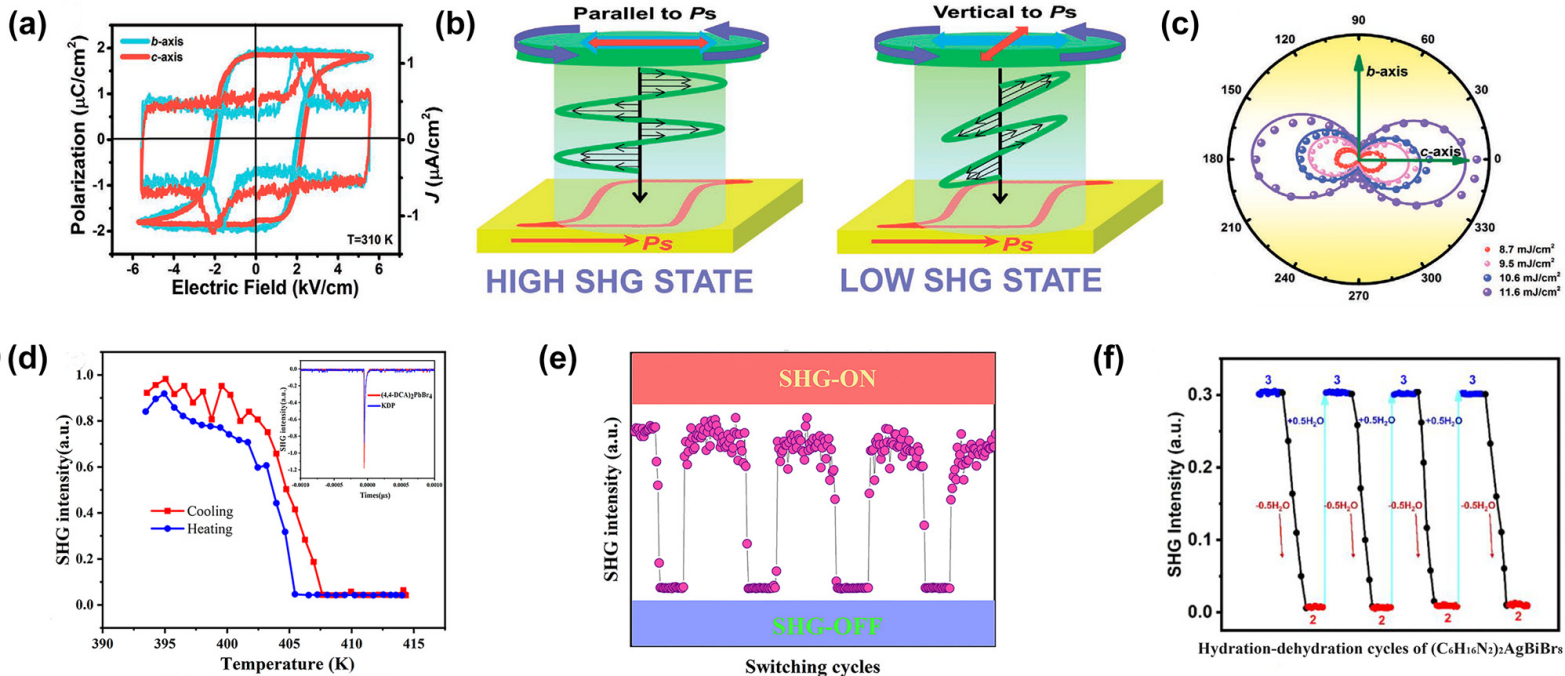
Tuning of SHG in 2D perovskites by external stimuli. (a) P-E hysteresis loops and related current density (J-E) traces collected along the *b*- and *c*-axes directions, revealing the biaxial characteristic of (IA)_2_(EA)_2_Pb_3_Br_10_ 2D perovskite. (b) Schematic illustration for tuning SHG properties of (IA)_2_(EA)_2_Pb_3_Br_10_ under the polarized light. (c) Tunable SHG results as a function of polarization angle under different laser energy for (IA)_2_(EA)_2_Pb_3_Br_10_ 2D perovskite. (d) Temperature dependent SHG intensity for (4, 4-DCA)_2_PbBr_4_ 2D perovskite. (e) Multiple cycle SHG switch for (4, 4-DCA)_2_PbBr_4_ 2D perovskite. (f) SHG signals in hydration-dehydration cycles between (C_6_H_16_N_2_)_2_AgBiBr_8_ and (C_6_H_16_N_2_)_2_AgBiBr_8_·H_2_O. (a), (b), (c) are reprinted with permission from [110]. Copyright 2021, John Wiley and Sons. (d), (e) are reprinted with permission from [122]. Copyright 2023, American Chemical Society. (f) is reprinted with permission from [116]. Copyright 2022, American Chemical Society.

Several works [110], [121], [122], [126] have demonstrated switching of the SHG signal through a reversible thermal phase transition. Yingjie Zhao et al. realized a reversible SHG switch without material degradation based on the transition between glassy and crystalline states in two-dimensional arrays of chiral (R-NPB)_2_PbBr_4_ perovskite microwires [121]. Ming-Yang Wang et al. also demonstrated temperature control of the SHG signal with minimal loss during a reversible phase transition process near the high Curie temperature (Tc ∼ 409 K) for polar 2D perovskite (4, 4-DCA)_2_PbBr_4_ (Figure 10(d) and (e)) [122]. The combination of efficient SHG with reversible phase transitions and relative durability makes 2D perovskites promising materials for temperature sensors.

#### 3.3.2 Electric field-induced switching

Another way to tune the SH signal is to modulate the material properties with an electric field. Ferroelectricity, which is one of the most important properties of materials, is inseparable from asymmetry and is usually accompanied by second-order NLO effects [129]. Yu Ma et al. presented improper ferroelectric of 2D multilayered perovskite (IA)_2_(EA)_2_Pb_3_Br_10_ (where IA is isoamylammonium and EA is ethylammonium), which exhibits ferroelectricity with a high *T*_*c*_ = 371 K and biaxial *P*_*s*_ ≈ 2.2 μC cm^−2^ (Figure 10(a)) [110]. Authors demonstrated tuning of SHG response by applying the polarized light, closely involving with intrinsic in-plane electric polarization. The maximum SH response is achieved when the direction of polarized light is parallel to Ps, and the minimum appears in the vertical direction (Figure 10(b) and (c)). The strong SHG anisotropy provides an extremely high dichroism ratio of ≈12, while the biaxial nature of ferroelectricity provides an ideal platform for tuning SHG properties through the polarity switching.

#### 3.3.3 Water-induced switching

Additionally, SH modulation can be implemented in two-dimensional perovskite materials by other methods, such as the structural symmetry breaking induced by water molecules in two-dimensional (2D) double perovskite (C_6_H_16_N_2_)_2_AgBiBr_8_ presented by Jiang-Feng Hong et al. [116]. Authors demonstrated reversible conversion of centric (C_6_H_16_N_2_)_2_AgBiBr_8_ with complete absence of SHG activity to acentric (C_6_H_16_N_2_)_2_AgBiBr_8_·0.5H_2_O with *χ* ^(2)^ = 0.51 pm/V (Figure 10(f)).

Basing on the previous examples, one can conclude that the high values of second-order nonlinear susceptibility, as well as the ability to widely tune SH properties depending on composition and external stimulation, make 2D perovskites excellent materials for nonlinear optical applications such as sensing, imaging, polarization-based optoelectronic devices, ultrafast modulators, etc.

## 4 Nanophotonics for SHG and manipulation

**Figure 11:**
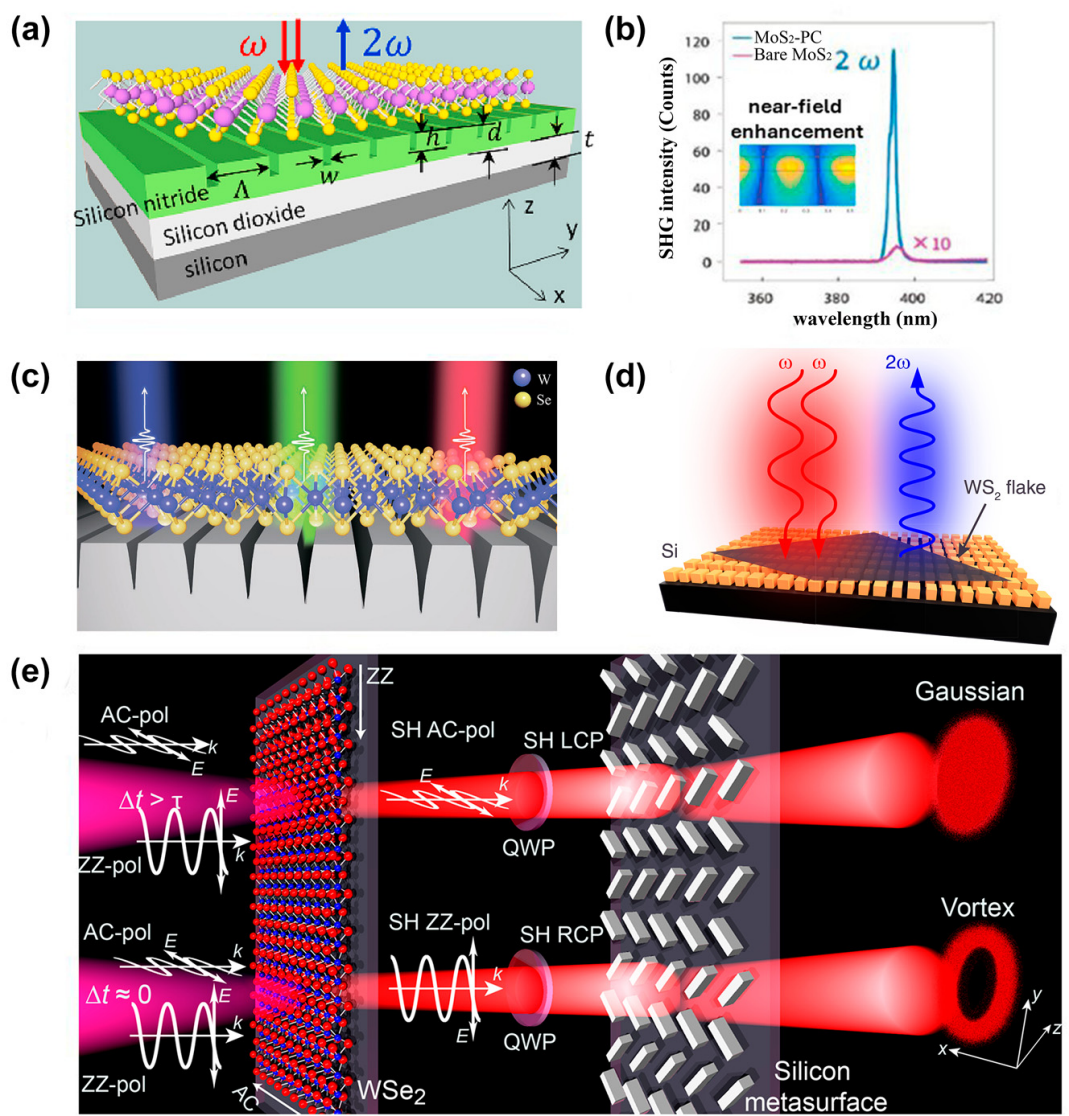
Nanophotonics for SHG and manipulation. (a) A schematic of second harmonic wave generation from a MoS_2_ monolayer placed on the top of a photonic crystal (PC). (b) SHG Spectrum profile of bare MoS_2_ and MoS_2_ on the PC. The spectrum from bare MoS_2_ is manually magnified by 10 times for better visibility. Inset: calculated near-field distribution on the resonant mode. Reproduced with permission [138]. Copyright 2020, Elsevier Publishing Group. (c) Design of the broadband tunable metasurface integrated with a 1L WSe_2_. Reproduced with permission [139]. Copyright 2021, Wiley-VCH GmbH. (d) Schematic of SHG from a 1L WS_2_ placed on top of a Si metasurface composed of a square array of bar pairs. Adapted with permission [140]. Copyright 2020, American Chemical Society. (e) Schematic representation of the operating principle of the cascaded TMD-metasurface structure for ultrafast wavefront shaping [141].

Even though the nonlinear optical response of 2D materials per unit thickness is exceptionally strong, the brief interaction between light and matter poses a significant challenge for achieving high nonlinear conversion efficiency in technological applications. Graphene and transition metal dichalcogenides (TMDs) are easily combined with various photonic and nanostructures, such as fibers [130], [131], waveguides [132]–[135], microrings [136], and nanowires [137] as well as in such planar systems as photonic crystals and metasurfaces offering a valuable means to boost light–matter interactions (see Figure 11). This section outlines potential strategies to tackle this challenge and highlights recent developments in this area with the help of nanophotonics and meta-optics.

In one of the first works [138], a photonic crystal supporting 170-folds SHG enhancement on monolayer MoS_2_ with a 1D photonic crystal compared to a bare monolayer MoS_2_ on SiO_2_ was demonstrated (Figure 11(a) and (b)). However, currently, wider used approach to enhance light–matter interaction in 2D materials is to combine them with nanostructured substrates such as resonant dielectric and plasmonic metasurfaces [138], [140], [142]–[145]. By gradually tuning the groove depth in a plasmonic metasurface, an enhanced SHG signal in the entire visible range was obtained [139] (Figure 11(c)). Indeed, plasmonic nanostructures and metasurfaces have also been used to enhance SHG in TMDs [146]. Shi et al. achieved a ≈400-fold enhancement of SHG in a monolayer WS_2_ incorporated onto a silver nanogroove grating that was finely tuned to match the resonant energy of the C exciton [146]. A three orders of magnitude SHG enhancement was observed in a monolayer WS_2_ transferred onto a gold film with sub-20 nm-wide trenches [147], as well as in a monolayer MoS_2_ on a suspended silver film patterned with a square nanohole array [148]. These hybrid samples exhibited modified SHG patterns due to directional enhancement and efficient polarization modulation. In the work [149], an elaborate metasurface of a monolayer WS_2_ on a plasmonic vortex metalens, which not only enhanced SHG but also generated a giant SHG circular dichroism was developed.

Recently, high-Q all-dielectric metasurfaces were also applied for SHG enhancement from 2D materials. In the work [140], it was demonstrated a three orders of magnitude second harmonic generation (SHG) enhancement in transition metal dichalcogenides (TMDs) by combining a monolayer of WS_2_ with an engineered asymmetric silicon substrate that supports high-Q modes (Figure 11(d)). Löchner et al. achieved a 35-fold SHG conversion efficiency enhancement by combining a monolayer TMD with a metasurface of periodic arrays of silicon nanoresonators that support a Fano resonance [145].

In addition to metasurfaces, an alternative approach for enhancing second harmonic generation (SHG) using localized plasmons was proposed in the work [150]. They achieved approximately a 300-fold enhancement in a monolayer (1L) WS_2_ coupled to a plasmonic nanocavity composed of silver nanocubes (with a length of approximately 75 nm) on a silver film, separated by a 10 nm Al_2_O_3_ layer. The enhancement in SHG was driven by the amplification of the local field, with theoretical analysis indicating that the enhancement is proportional to the square of the local field intensity within the nanocavity under the interaction between the SH dipole in the 1L TMD and the electric quadrupole in the nanocavity.

In the work [141], the authors showcased the ultrafast manipulation of wavefronts through an advanced meta-optical setup involving a monolayer TMD, a quarter-wave plate, and a silicon metasurface (Figure 11(e)). Through detailed spatially resolved pump-probe experiments, they illustrate the feasibility of achieving rapid transformations such as second harmonic beam deflection, transitioning from Gaussian to vortex beams, and altering topological charges on a femtosecond time scale. Notably, leveraging the polarization-selective nature of metasurfaces to encode complex spatial phase profiles opens up possibilities for seamless switching between diverse wavefronts with customizable intensity profiles and ultrafast dynamics. These findings hold promise for driving innovation in high-speed communications, remote sensing, ultrafast optics, and holographic applications.

## 5 Conclusions and future outlook

To summarize, recent advancements in the effective characterization of the SHG properties of 2D metal chalcogenide and perovskite atomically thin crystals has been presented and discussed. A unified theoretical framework of nonlinear optics for P-SHG measurements, that is applied to the latest literature reports, has been particularly demonstrated. It can be concluded that all-optical minimally invasive SHG and P-SHG are proven to be a powerful tool to unveil the physical properties of such 2D crystals and probe crystal orientation, number of layers, various types of defects, grain boundary, interlayer stacking, stacking angle, in-plane anisotropy, valley polarization and strain. Despite the rapid progress of SHG applied in 2D crystals, there is still a lot of room for further exploring the physics of 2D materials. Among potential capabilities, P-SHG could be potentially exploited to precisely determine the susceptibility tensor elements in 2D crystals. Furthermore, P-SHG can be combined with pump–probe spectroscopy in order to probe the ultrafast carrier dynamics under non-linear excitation conditions. In addition, P-SHG could provide a new platform to explore the unexplored physics behind valley polarization and dark excitonic phenomena.

Furthermore, first attempts to tune and control the SHG properties via nanophotonic schemes are presented and discussed. The overview of modern nanophotonic approaches has shown a great potential for enhancement and manipulation of SH from 2D materials like TMDs. Indeed, intensity, polarization, and beam-shape control has been demonstrated during last several years. However, this powerful nanophotonic platform has not been applied for SHG from 2D halide perovskites yet. There were reports on odd (3^rd^ and 5^th^) harmonics generation from perovskite metasurfaces [151], as well as multiphoton photoluminescence enhancement [152]–[154]. On the other hand, halide perovskite family of materials with second-order nonlinearity can be also nanostructured or integrated with non-perovskite metasurfaces to enhance or tailor their SHG. Recently, strong spectrally tunable SHG signal enhancement was demonstrated for lead-free germanium halide perovskite nanoparticles via coupling of incident light with Mie-type resonances [155]. For multilayer TMDs materials, SHG similarly can be improved or controlled by their direct patterning [156]. Therefore, we envision that the direct nanopatterning of 2D materials for SHG manipulation is an emerging and rapidly developing field of research during next several years.

## References

[1] Franken P. A., Hill A. E., Peters C. W., Weinreich G. (1961). Generation of optical harmonics. *Phys. Rev. Lett.*.

[2] Fu Y. (2024). Optical second harmonic generation of low-dimensional semiconductor materials. *Nanomaterials*.

[3] Wang Y., Xiao J., Yang S., Zhang X. (2019). Second harmonic generation spectroscopy on two-dimensional materials. *Opt. Mater. Express*.

[4] Ma H. (2020). Rich information on 2D materials revealed by optical second harmonic generation. *Nanoscale*.

[5] Zhou L. (2020). Nonlinear optical characterization of 2D materials. *Nanomaterials*.

[6] Shi J., Feng S., He P., Fu Y., Zhang X. (2023). Nonlinear optical properties from engineered 2D materials. *Molecules*.

[7] Xie Z., Zhao T., Yu X., Wang J. (2024). Nonlinear optical properties of 2D materials and their applications. *Small*.

[8] Huang W., Xiao Y., Xia F., Chen X., Zhai T. (2024). Second harmonic generation control in 2D layered materials: status and outlook. *Adv. Funct. Mater.*.

[9] Autere A., Jussila H., Dai Y., Wang Y., Lipsanen H., Sun Z. (2018). Nonlinear optics with 2D layered materials. *Adv. Mater.*.

[10] Liu X., Guo Q., Qiu J. (2017). Emerging low-dimensional materials for nonlinear optics and ultrafast photonics. *Adv. Mater.*.

[11] Yu S., Wu X., Wang Y., Guo X., Tong L. (2017). 2D materials for optical modulation: challenges and opportunities. *Adv. Mater.*.

[12] You J. W., Bongu S. R., Bao Q., Panoiu N. (2018). Nonlinear optical properties and applications of 2D materials: theoretical and experimental aspects. *Nanophotonics*.

[13] Sun Z., Martinez A., Wang F. (2016). Optical modulators with 2D layered materials. *Nat. Photonics*.

[14] Guo B., Xiao Q.-L., Wang S.-H., Zhang H. (2019). 2D layered materials: synthesis, nonlinear optical properties, and device applications. *Laser Photonics Rev.*.

[15] Bonaccorso F., Sun Z., Hasan T., Ferrari A. C. (2010). Graphene photonics and optoelectronics. *Nat. Photonics*.

[16] Sun Z. (2019). Graphene mode-locked ultrafast laser. *ACS Nano*.

[17] Martinez A., Sun Z. (2013). Nanotube and graphene saturable absorbers for fibre lasers. *Nat. Photonics*.

[18] Wang G. (2019). Saturable absorption in 2D nanomaterials and related photonic devices. *Laser Photonics Rev.*.

[19] Lim G. K. (2011). Giant broadband nonlinear optical absorption response in dispersed graphene single sheets. *Nat. Photonics*.

[20] Xiao J., Zhao M., Wang Y., Zhang X. (2017). Excitons in atomically thin 2D semiconductors and their applications. *Nanophotonics*.

[21] Krasnok A., Lepeshov S., Alú A. (2018). Nanophotonics with 2D transition metal dichalcogenides [invited]. *Opt. Express*.

[22] Cotrufo M., Sun L., Choi J., Alù A., Li X. (2019). Enhancing functionalities of atomically thin semiconductors with plasmonic nanostructures. *Nanophotonics*.

[23] Sortino L. (2019). Enhanced light-matter interaction in an atomically thin semiconductor coupled with dielectric nano-antennas. *Nat. Commun.*.

[24] Sun L. (2019). Separation of valley excitons in a MoS_2_ monolayer using a subwavelength Asymmetric groove array. *Nat. Photonics*.

[25] Seyler K. L. (2015). Electrical control of second-harmonic generation in a WSe_2_ monolayer transistor. *Nat. Nanotechnol.*.

[26] Merkl P. (2020). Twist-tailoring coulomb correlations in van Der waals homobilayers. *Nat. Commun.*.

[27] Chen J. H., Tan J., Wu G. X., Zhang X. J., Xu F., Lu Y. Q. (2019). Tunable and enhanced light emission in hybrid WS_2_ – optical – Fiber Nanowire structures. *Light: Sci. Appl.*.

[28] Wang Y. (2017). Structural phase transition in monolayer MoTe2 driven by electrostatic doping. *Nature*.

[29] Lin X. (2018). Two-dimensional pyramid-likeWS2 layered structures for highly efficient edge second-harmonic generation. *ACS Nano*.

[30] Novoselov K. S. (2004). Electric field effect in atomically thin carbon films. *Science*.

[31] Sarkar A. S., Stratakis E. (2020). Recent advances in 2D metal monochalcogenides. *Advanced Science*.

[32] Xia F., Wang H., Hwang J. C. M., Neto A. H. C., Yang L. (2019). Black phosphorus and its isoelectronic materials. *Nat. Rev. Phys.*.

[33] Li L. (2019). The rise of two-dimensional materials for advanced optoelectronic applications. *InfoMat*.

[34] Gomes L. C., Carvalho A. (2015). Phosphorene analogues: isoelectronic two-dimensional group-IV monochalcogenides with orthorhombic structure. *Phys. Rev. B*.

[35] Liu H. (2014). Phosphorene: an unexplored 2D semiconductor with a high hole mobility. *ACS Nano*.

[36] Carvalho A. (2016). Phosphorene: from theory to applications. *Nat. Rev. Mater.*.

[37] Tian Z., Guo C., Zhao M., Li R., Xue J. (2017). Two-dimensional SnS: a phosphorene analogue with strong in-plane electronic anisotropy. *ACS Nano*.

[38] Yang Y. (2019). In-plane optical anisotropy of low-symmetry 2D GeSe. *Adv. Opt. Mater.*.

[39] Radisavljevic B., Radenovic A., Brivio J., Giacometti V., Kis A. (2011). Single-layer MoS_2_ transistors. *Nat. Nanotechnol.*.

[40] Cao T. (2012). Valley-selective circular dichroism of monolayer molybdenum disulphide. *Phys. Rev. B*.

[41] Zeng H., Dai J., Yao W., Xiao D., Cui X. (2012). Valley polarization in MoS2 monolayers by optical pumping. *Nat. Nanotechnol.*.

[42] Mak K. F., McGill K. L., Park J., McEuen P. L. (2014). The valley Hall effect in MoS_2_ transistors. *Science*.

[43] Zhang X., Tan Q. H., Wu J. B., Shi W., Tan P. H. (2016). Review on the Raman spectroscopy of different types of layered materials. *Nanoscale*.

[44] Zhao W. (2013). Evolution of electronic structure in atomically thin sheets of WS2 and WSe2. *ACS Nano*.

[45] Li Y. (2014). Measurement of the optical dielectric function of monolayer transition-metal dichalcogenides: MoS2, MoSe2, WS2, and WSe2. *Phys. Rev. B*.

[46] Boyd R. W. (2008). *Nonlinear Optics*.

[47] Shen Y. R. (1989). Surface properties probed by second-harmonic and sum-frequency generation. *Nature*.

[48] Yin X. (2014). Edge nonlinear optics on a MoS_2_ atomic monolayer. *Science*.

[49] Kumar N. (2013). Second harmonic microscopy of monolayer MoS_2_. *Phys. Rev. B: Condens. Matter Mater. Phys.*.

[50] Wang G. (2015). Giant enhancement of the optical second-harmonic emission of WSe2 monolayers by laser excitation at exciton resonances. *Phys. Rev. Lett.*.

[51] Ning T., Zhao L., Huo Y., Cai Y., Ren Y. (2023). Giant enhancement of second harmonic generation from monolayer 2D materials placed on photonic moiré superlattice. *Nanophotonics*.

[52] Vianna P. G., Almeida A. S., Gerosa R. M., Bahamon D. A., de Matos C. J. S. (2021). Second-harmonic generation enhancement in monolayer transition-metal dichalcogenides by using an epsilon-near-zero substrate. *Nanoscale Adv.*.

[53] Zhang C.-C. (2024). Plasmon-enhanced second harmonic generation of metal nanostructures. *Nanoscale*.

[54] Aghigh A., Bancelin S., Rivard M., Pinsard M., Ibrahim H., Légaré F. (2023). Second harmonic generation microscopy: a powerful tool for bio-imaging. *Biophys. Rev.*.

[55] Haussühl S. (2007). *Physical Properties Roof Crystals*.

[56] McIver J. W., Hsieh D., Steinberg H., Jarillo-Herrero P., Gedik N. (2011). Control over topological insulator photocurrents with light polarization. *Nat. Nanotechnol.*.

[57] Malard L. M. (2013). Observation of intense second harmonic generation from MoS_2_ atomic crystals. *Phys. Rev. B: Condens. Matter Mater. Phys.*.

[58] Li Y. (2013). Probing symmetry properties of few-layer MoS2 and h-BN by optical second-harmonic generation. *Nano Lett.*.

[59] Psilodimitrakopoulos S. Ultrahigh-resolution nonlinear optical imaging of the armchair orientation in 2D transition metal dichalcogenides. *Light: Sci. Appl.*.

[60] Zhao M. (2016). Atomically phase-matched second-harmonic generation in a 2D crystal. *Light: Sci. Appl.*.

[61] Song Y., Hu S., Lin M. L., Gan X., Tan P. H., Zhao J. (2018). Extraordinary second harmonic generation in ReS_2_ atomic crystals. *ACS Photonics*.

[62] Maragkakis G. M. (2019). Imaging the crystal orientation of 2D transition metal dichalcogenides using polarization-resolved second-harmonic generation. *Opto-Electron. Adv.*.

[63] David S. N. (2015). Rapid, all-optical crystal orientation imaging of two-dimensional transition metal dichalcogenide monolayers. *Appl. Phys. Lett.*.

[64] Karvonen L. (2017). Rapid visualization of grain boundaries in monolayer MoS2 by multiphoton microscopy. *Nat. Commun.*.

[65] Hsu W. T. (2014). Second harmonic generation from artificially stacked transition metal dichalcogenide twisted bilayers. *ACS Nano*.

[66] Psilodimitrakopoulos S. (2019). Twist angle mapping in layered WS_2_ by polarization-resolved second harmonic generation. *Sci. Rep.*.

[67] Maragkakis G. M. (2022). Nonlinear optical imaging of in-plane anisotropy in two-dimensional SnS. *Adv. Opt. Mater.*.

[68] Mouchliadis L. (2021). Probing valley population imbalance in transition metal dichalcogenides via temperature-dependent second harmonic generation imaging. *npj 2D Mater. Appl.*.

[69] Ho Y. W. (2020). Measuring valley polarization in transition metal dichalcogenides with second-harmonic spectroscopy. *ACS Photonics*.

[70] Liang J. (2017). Monitoring local strain vector in atomic-layered MoSe_2_ by second-harmonic generation. *Nano Lett.*.

[71] Mennel L. Optical imaging of strain in two-dimensional crystals. *Nat. Commun.*.

[72] Geim A. K., Grigorieva I. V. (2013). Van der Waals heterostructures. *Nature*.

[73] Novoselov K. S., Mishchenko A., Carvalho A., Castro Neto A. H. (2016). 2D materials and van der Waals heterostructures. *Science*.

[74] Wang X., Xia F. (2015). Van der Waals heterostructures: Stacked 2D materials shed light. *Nat. Mater.*.

[75] Liu K. (2014). Evolution of interlayer coupling in twisted MoS2 bilayers. *Nat. Commun.*.

[76] Liao M. (2022). Ultra-low friction and edge-pinning effect in large-lattice-mismatch van der Waals heterostructures. *Nat. Mater.*.

[77] Kim K. (2012). Raman spectroscopy study of rotated double-layer graphene: misorientation-angle dependence of electronic structure. *Phys. Rev. Lett.*.

[78] Kou L., Frauenheim T., Chen C. (2013). Nanoscale multilayer transition-metal dichalcogenide heterostructures: band gap modulation by interfacial strain and spontaneous polarization. *J. Phys. Chem. Lett.*.

[79] Psilodimitrakopoulos S. Optical versus electron diffraction imaging of Twist-angle in 2D transition metal dichalcogenide bilayer superlattices,” *npj 2D Mater*. *Appl.*.

[80] Zu R. (2024). Optical second harmonic generation in anisotropic multilayers with complete multireflection of linear and nonlinear waves using ♯SHAARP.ml package. *npj Comput. Mater.*.

[81] Psilodimitrakopoulos S. Real-time spatially resolved determination of twist angle in transition metal dichalcogenide heterobilayers. *2D Materials*.

[82] Higashitarumizu N. Purely in-plane ferroelectricity in monolayer SnS at room temperature. *Nat. Commun.*.

[83] Wang H., Qian X. (2017). Giant optical second harmonic generation in two-dimensional multiferroics. *Nano Lett.*.

[84] Panday S. R., Fregoso B. M. (2017). Strong second harmonic generation in two-dimensional ferroelectric IV-monochalcogenides. *J. Phys.: Condens. Matter*.

[85] Zhu M. (2021). Efficient and anisotropic second harmonic generation in few-layer SnS film. *Adv. Opt. Mater.*.

[86] Beams R. (2016). Characterization of few-layer 1T’ MoTe2 by polarization-resolved second harmonic generation and Raman scattering. *ACS Nano*.

[87] Akinwande D., Petrone N., Hone J. (2014). Two-dimensional flexible nanoelectronics. *Nat. Commun.*.

[88] Mak K. F., Shan J. (2016). Photonics and optoelectronics of 2D semiconductor transition metal dichalcogenides. *Nat. Photonics*.

[89] Yu J. R. S. H. (2016). Valleytronics in 2D materials. *Nat. Rev. Mater.*.

[90] Hipolito F., Pereira V. M. (2017). Second harmonic spectroscopy to optically detect valley polarization in 2D materials. *Two-Dimens. Mater.*.

[91] Herrmann P. (2023). Nonlinear all-optical coherent generation and read-out of valleys in atomically thin semiconductors. *Small*.

[92] Golub L. E., Tarasenko S. A. (2014). Valley polarization induced second harmonic generation in graphene. *Phys. Rev. B*.

[93] Wehling T. O., Huber A., Lichtenstein A. I., Katsnelson M. I. (2015). Probing of valley polarization in graphene via optical second-harmonic generation. *Phys. Rev. B*.

[94] Goryca M. (2019). Detection of thermodynamic ‘valley noise’ in monolayer semiconductors: access to intrinsic valley relaxation time scales. *Sci. Adv.*.

[95] Feng J., Qian X., Huang C.-W., Li J. (2012). Strain-engineered artificial atom as a broad-spectrum solar energy funnel. *Nat. Photonics*.

[96] Akinwande D., Nicholas P., Hone J. (2014). Two-dimensional flexible nanoelectronics. *Nat. Commun.*.

[97] Akkerman Q. A., Manna L. (2020). What defines a halide perovskite?. *ACS Energy Lett.*.

[98] Mao L., Wu Y., Stoumpos C. C., Wasielewski M. R., Kanatzidis M. G. (2017). White-light emission and structural distortion in new corrugated two-dimensional lead bromide perovskites. *J. Am. Chem. Soc.*.

[99] Pengfei L. (2017). Two-dimensional ch3nh3pbi3 perovskite nanosheets for ultrafast pulsed fiber lasers. *ACS Appl. Mater. Interfaces*.

[100] Abdelwahab I. (2019). Giant and tunable optical nonlinearity in single- crystalline 2d perovskites due to excitonic and plasma effects. *Adv. Mater.*.

[101] Mercier N. (2019). Hybrid halide perovskites: discussions on terminology and materials. *Angew. Chem., Int. Ed.*.

[102] Soe C. M. M. (2017). New type of 2d perovskites with alternating cations in the interlayer space,(c (nh2) 3)(ch3nh3) n pb n i3 n+ 1: structure, properties, and photovoltaic performance. *J. Am. Chem. Soc.*.

[103] Yuan C., Li X., Semin S., Feng Y., Rasing T., Xu J. (2018). Chiral lead halide perovskite nanowires for second-order nonlinear optics. *Nano Lett.*.

[104] Blancon J.-C., Even J., Stoumpos C. C., Kanatzidis M. G., Mohite A. D. (2020). Semiconductor physics of organic–inorganic 2d halide perovskites. *Nat. Nanotechnol.*.

[105] Wei W.-J. (2019). Regulating second-harmonic generation by van der waals interactions in two-dimensional lead halide perovskite nanosheets. *J. Am. Chem. Soc.*.

[106] Wu Z. (2018). Alloying n-butylamine into cspbbr3 to give a two-dimensional bilay-ered perovskite ferroelectric material. *Angew. Chem., Int. Ed.*.

[107] Li L. (2019). Two-dimensional hybrid perovskite-type ferroelectric for highly polarization-sensitive shortwave photodetection. *J. Am. Chem. Soc.*.

[108] Han S. (2019). High-temperature antiferroelectric of lead iodide hybrid perovskites. *J. Am. Chem. Soc.*.

[109] Yang C.-K. (2019). The first 2d homochiral lead iodide perovskite ferroelectrics:[r-and s-1-(4-chlorophenyl) ethylammonium] 2pbi4. *Adv. Mater.*.

[110] Ma Y. (2021). The first improper ferroelectric of 2d multilayered hybrid per-ovskite enabling strong tunable polarization-directed second harmonic generation effect. *Adv. Funct. Mater.*.

[111] Tang L. (2023). Photoexcited ultraviolet-to-infrared (ii) pyroelectricity in a2d ferroelectric perovskite driving broadband self-powered photoactivities. *Adv. Funct. Mater.*.

[112] Trujillo-Hern ández K. (2020). Chirality control in white- light emitting 2d perovskites. *J. Mater. Chem. C*.

[113] Chakraborty R. (2023). Rational design of non-centrosymmetric hybrid halide perovskites. *J. Am. Chem. Soc.*.

[114] Chen C. (2023). Two-dimensional hybrid dion–jacobson germanium halide perovskites. *Chem. Mater.*.

[115] Shi P.-P. (2019). Two-dimensional organic–inorganic perovskite ferroelectric semiconductors with fluorinated aromatic spacers. *J. Am. Chem. Soc.*.

[116] Hong J.-F. (2022). Water-driven successive structural transformation in a two-dimensional (2d) lead-free hybrid double perovskite. *Inorg. Chem.*.

[117] Li X., Guan Y., Li X., Fu Y. (2022). Stereochemically active lone pairs and nonlinear optical proper- ties of two-dimensional multilayered tin and germanium iodide perovskites. *J. Am. Chem. Soc.*.

[118] Maczka M. (2021). [methylhydrazinium] 2pbbr4, a ferroelectric hybrid organic–inorganic perovskite with multiple nonlinear optical outputs. *Chem. Mater.*.

[119] Stoumpos C. C. (2016). Ruddlesden–popper hybrid lead iodide perovskite2d homologous semiconductors. *Chem. Mater.*.

[120] Zhao J. (2023). Chiral hybrid perovskites (r-/s-clpea) 4bi2i10 with enhanced chirality and spin–orbit coupling splitting for strong nonlinear optical circular dichroism and spin selectivity effects. *Chem. Mater.*.

[121] Zhao Y. (2022). Reversible phase transition for switchable second harmonic generation in 2d perovskite microwires. *SmartMat*.

[122] Wan M.-Y. (2023). Excellent switchable properties, broad-band emission, ferroelectricity, and high t c in a two-dimensional hybrid perovskite:(4, 4-dca) 2pbbr4 exploited by h/f substitution. *Inorg. Chem.*.

[123] Wang S. (2018). Highly efficient white-light emission in a polar two-dimensional hybrid perovskite. *Chem. Commun.*.

[124] Morrow D. J. (2020). Disentangling second harmonic genera- tion from multiphoton photoluminescence in halide perovskites using multidimensional harmonic generation. *J. Phys. Chem. Lett.*.

[125] Mihalyi-Koch W. (2023). Tuning structure and excitonic properties of 2d ruddlesden–popper germanium, tin, and lead iodide perovskites via interplay between cations. *J. Am. Chem. Soc.*.

[126] Fedoruk K. (2022). [methylhydrazinium]2pbcl4, a two-dimensional perovskite with polar and modulated phases. *Inorg. Chem.*.

[127] Fleming J. W. (2018). *Handbook of Optical Materials*.

[128] Li R. (2024). Perovskitizer tuning pb2+ 6s2 lone pair effects in hybrid perovskite halide enables multiaxial self-powered x-ray detection. *Adv. Funct. Mater.*.

[129] Han X., Zheng Y., Chai S., Chen S., Xu J. (2020). 2d organic-inorganic hybrid perovskite materials for nonlinear optics. *Nanophotonics*.

[130] An N. (2020). Electrically tunable four-wave-mixing in graphene heterogeneous fiber for individual gas molecule detection. *Nano Lett.*.

[131] Chen J.-H., Tan J., Wu G.-X., Zhang X.-J., Xu F., Lu Y.-Q. (2019). Tunable and enhanced light emission in hybrid WS_2_-optical-fiber-nanowire structures. *Light: Sci. Appl.*.

[132] Montanaro A. (2021). Optoelectronic mixing with high-frequency graphene transistors. *Nat. Commun.*.

[133] Alexander K., Savostianova N. A., Mikhailov S. A., Kuyken B., Van Thourhout D. (2017). Electri- cally tunable optical nonlinearities in graphene-covered sin waveguides characterized by four-wave mixing. *ACS Photonics*.

[134] Guo Q. (2020). Efficient frequency mixing of guided surface waves by atomically thin nonlinear crystals. *Nano Lett.*.

[135] Chen H. (2017). Enhanced second-harmonic generation from two- dimensional MoSe_2_ on a silicon waveguide. *Light: Sci. Appl.*.

[136] He J. (2021). Low-loss integrated nanophotonic circuits with layered semi- conductor materials. *Nano Lett.*.

[137] Li D. (2019). Anisotropic enhancement of second-harmonic generation in mono- layer and bilayer MoS_2_ by integrating with tio2 nanowires. *Nano Lett.*.

[138] Zhang Z., Zhang L., Gogna R., Chen Z., Deng H. (2020). Large enhancement of second-harmonic generation in MoS2 by one dimensional photonic crystals. *Solid State Commun.*.

[139] Ding Y. (2021). Second harmonic generation covering the entire visible range from a 2d material–plasmon hybrid metasurface. *Adv. Opt. Mater.*.

[140] Bernhardt N. (2020). Quasi-bic resonant enhancement of second- harmonic generation in WS_2_ monolayers. *Nano Lett.*.

[141] Sinelnik A. (2024). Ultrafast all-optical second harmonic wavefront shap- ing. *Nat. Commun.*.

[142] Yi F. (2016). Optomechanical enhancement of doubly resonant 2d optical nonlinearity. *Nano Lett.*.

[143] Day J. K., Chung M.-H., Lee Y.-H., Menon V. M. (2016). Microcavity enhanced second harmonic generation in 2d MoS_2_. *Opt. Mater. Express*.

[144] Chen B. (2020). Simultaneously enhanced linear and nonlinear photon generations from WS_2_ by using dielectric circular Bragg resonators. *Nanophotonics*.

[145] Löchner F. J. (2020). Hybrid dielectric metasurfaces for enhancing second- harmonic generation in chemical vapor deposition grown MoS_2_ monolayers. *ACS Photonics*.

[146] Shi J. (2018). Plasmonic enhancement and manipulation of optical non- linearity in monolayer tungsten disulfide. *Laser Photonics Rev.*.

[147] Wang Z. (2018). Selectively plasmon-enhanced second-harmonic generation from monolayer tungsten diselenide on flexible substrates. *ACS Nano*.

[148] Leng Q. (2021). Enhanced second-harmonic generation in monolayer MoS_2_ on sus- pended metallic nanostructures by plasmonic resonances. *Nanophotonics*.

[149] Guo W.-P. (2020). Chiral second-harmonic generation from monolayer WS_2_/aluminum plasmonic vortex metalens. *Nano Lett.*.

[150] Han X. (2020). Harmonic resonance enhanced second-harmonic generation in the monolayer WS_2_–Ag nanocavity. *ACS Photonics*.

[151] Tonkaev P., Koshelev K., Masharin M. A., Makarov S. V., Kruk S. S., Kivshar Y. (2023). Observation of enhanced generation of a fifth harmonic from halide perovskite nonlocal metasurfaces. *ACS Photonics*.

[152] Makarov S. V. (2017). Multifold emission enhancement in nanoim- printed hybrid perovskite metasurfaces. *ACS Photonics*.

[153] Fan Y. (2021). Enhanced multiphoton processes in perovskite metasurfaces. *Nano Lett.*.

[154] Fan Y. (2019). Resonance-enhanced three-photon luminesce via lead halide perovskite metasurfaces for optical encoding. *Nat. Commun.*.

[155] Ilin S. (2024). Lead‐free halide perovskite nanoparticles for up‐conversion lasing and efficient second harmonic generation. *Adv. Opt. Mater.*.

[156] Zograf G., Polyakov A. Y., Bancerek M., Antosiewicz T., Kucukoz B., Shegai T. (2023). Com- bining ultrahigh index with exceptional nonlinearity in resonant transition metal dichalcogenide nanodisks. ..

